# Micro-CT-assisted identification of the optimal time-window for antifibrotic treatment in a bleomycin mouse model of long-lasting pulmonary fibrosis

**DOI:** 10.1038/s41598-024-65030-3

**Published:** 2024-06-26

**Authors:** Martina Buccardi, Andrea Grandi, Erica Ferrini, Davide Buseghin, Gino Villetti, Maurizio Civelli, Nicola Sverzellati, Andrea Aliverti, Francesca Pennati, Franco Fabio Stellari

**Affiliations:** 1 Department of Mathematical, Physical and Computer Sciences, University of Parma, Parma, Italy; 2 Experimental Pharmacology and Translational Science Department, Chiesi Farmaceutici S.P.A, 43122 Parma, Italy; 3 Department of Veterinary Science, University of Parma, Parma, Italy; 4 Dipartimento di Elettronica, Informazione e Bioingegneria, Politecnico di Milano, Milan, Italy; 5 ANTHEM (AdvaNced Technologies for Human-centrEd Medicine), Spoke 3, Milan, Italy; 6 Department of Medicine and Surgery, University of Parma, Parma, Italy

**Keywords:** Bleomycin model, Lung fibrosis, IPF, Drug discovery, Micro-computed tomography, Functional measurements, Nintedanib treatment, Drug discovery, Preclinical research, Experimental models of disease

## Abstract

Idiopathic Pulmonary Fibrosis (IPF) is a debilitating and fatal lung disease characterized by the excessive formation of scar tissue and decline of lung function. Despite extensive research, only two FDA-approved drugs exist for IPF, with limited efficacy and relevant side effects. Thus, there is an urgent need for new effective therapies, whose discovery strongly relies on IPF animal models. Despite some limitations, the Bleomycin (BLM)-induced lung fibrosis mouse model is widely used for antifibrotic drug discovery and for investigating disease pathogenesis. The initial acute inflammation triggered by BLM instillation and the spontaneous fibrosis resolution that occurs after 3 weeks are the major drawbacks of this system. In the present study, we applied micro-CT technology to a longer-lasting, triple BLM administration fibrosis mouse model to define the best time-window for Nintedanib (NINT) treatment. Two different treatment regimens were examined, with a daily NINT administration from day 7 to 28 (NINT 7–28), and from day 14 to 28 (NINT 14–28). For the first time, we automatically derived both morphological and functional readouts from longitudinal micro-CT. NINT 14–28 showed significant effects on morphological parameters after just 1 week of treatment, while no modulations of these biomarkers were observed during the preceding 7–14-days period, likely due to persistent inflammation. Micro-CT morphological data evaluated on day 28 were confirmed by lung histology and bronchoalveolar lavage fluid (BALF) cells; Once again, the NINT 7–21 regimen did not provide substantial benefits over the NINT 14–28. Interestingly, both NINT treatments failed to improve micro-CT-derived functional parameters. Altogether, our findings support the need for optimized protocols in preclinical studies to expedite the drug discovery process for antifibrotic agents. This study represents a significant advancement in pulmonary fibrosis animal modeling and antifibrotic treatment understanding, with the potential for improved translatability through the concurrent structural–functional analysis offered by longitudinal micro-CT.

## Introduction

Idiopathic Pulmonary Fibrosis (IPF) is a progressive and highly debilitating lung disease characterized by the accumulation of fibrotic tissue, ultimately leading to respiratory failure. Despite extensive research efforts directed toward comprehending the pathogenesis of IPF and developing innovative therapeutic strategies, there are still unmet needs in the pharmacological management of this condition. FDA-approved pharmacological treatments for IPF are currently limited to Nintedanib and Pirfenidone. Even though both drugs have demonstrated efficacy in reducing the decline in Forced Vital Capacity (FVC)^1^, especially in cases characterized by mild to moderate severity of fibrosis, neither is capable of halting disease progression^2^. Moreover, they come with significant side effects^3^ the severity of which varies among patients. Lung transplantation often remains the only option for IPF patients^4^. There is thus a pressing need for the development of alternative anti-fibrotic therapeutics that can effectively target the causative mechanisms of fibrosis without compromising patient well-being^5,6^.

While many compounds demonstrate efficacy in preclinical trials and advance to the clinical phase, the vast majority ultimately fail and never reach the market. In this challenging landscape of drug discovery^7^, the availability of reliable animal models becomes crucial for the discovery and preclinical evaluation of new antifibrotic drugs.

The Bleomycin (BLM)-induced lung fibrosis model is a well-established and widely used experimental system for studying IPF pathophysiology as well as for the discovery and testing of new antifibrotic drugs. While BLM instillation induces a fairly strong fibrotic response displaying the same histological hallmarks as those observed in IPF patients^8^, it also presents some serious drawbacks, such as an initial acute inflammation immediately following BLM administration and the spontaneous resolution of fibrosis 3 weeks after BLM.

As previously pointed out^9^, the timing of therapeutic interventions in preclinical models of IPF is a crucial parameter that can affect the discovery of new candidate drugs as well as the development of novel prototype lead compounds. To optimize the efficiency of the drug discovery process, it is crucial to assess antifibrotic efficacy effectively while avoiding possible confounding effects; hence, candidate drugs should not affect early inflammation, yet be allowed a time of action long enough for target engagement. Given the initial inflammatory response induced by BLM treatment and the spontaneous fibrosis resolution that occurs in the third week, the temporal window for therapeutic interventions is currently limited to 2 weeks (from day 7 to day 21).

To overcome these limitations, we have recently developed a longer-lasting mouse model of pulmonary fibrosis (LLMMPF) based on a triple BLM treatment, which induces, pulmonary fibrosis lasting for up to 28 days^10^. With this model, the therapeutic window may thus be extended by up to 3 weeks, from day 7 to day 28, allowing drug screening in the later, and likely more relevant stages of fibrosis. An alternative option is to limit therapeutic interventions to the day 14–28 window, thus bypassing the early acute inflammatory phase.

The aim of the present study was to determine, in the LLMMPF, the optimal time window for Nintedanib treatment -a human use-approved antifibrotic and the reference drug for preclinical studies. In particular, we sought to compare the therapeutic efficacy of NINT administered within the 7–28 and 14–28 time-windows, using micro-CT-derived morphological and functional biomarkers^11^ as well as histological analyses to assess lung fibrosis progression.

## Materials and methods

### Experimental animals

The experiments were conducted on 7–8-weeks-old male C57Bl/6 mice purchased from Envigo (San Pietro al Natisone, Udine, Italy). Animals were housed in groups of five per cage under standard conditions in our animal facility. Upon delivery, the animals were acclimatized to our local vivarium conditions for 7–10 days (room temperature: 20–24 °C; relative humidity: 40–70%; 12 h light–dark cycle; food and water ad libitum). All necessary measures were taken to minimize pain or discomfort to the animals, and a designated veterinarian or trained technicians evaluated pain levels daily using a Visual Analogue Scale (VAS) ranging from 0 to 10. Humane endpoints were defined as the presence of dyspnea, body weight loss ≥ 20%, and VAS ≥ 6.

### Ethics

All experiments were carried out in accordance with the intramural animal welfare practices for animal experimentation of Chiesi Farmaceutici S.p.A. and complied with the European Directive 2010/63/UE, Italian D.Lgs 26/2014, the revised “Guide for the Care and Use of Laboratory Animals” (National Research Council Committee, US, 2011)^12^ and in accordance with ARRIVE guidelines^13^. The study was conducted in an AAALAC (Association for Assessment and Accreditation for Laboratory Animal Care) certified facility at Chiesi Farmaceutici; all the experimental animal procedures used in the study were approved by the Italian Ministry of Health (protocol number n° 809/2020-PR) and by the internal AWB (Animal Welfare Body).

### Experimental protocol

A total of 23 mice were included in this study. Pulmonary fibrosis was induced by administering bleomycin hydrochloride (Baxter) diluted in 50 μL saline via a triple (day 0, 2, 4) oropharyngeal aspiration (OA) under 2.5% isoflurane anesthesia, following previously described methods^10^. The negative control group received a triple OA of saline (SAL). Subsequently, the BLM-treated mice were randomized into three groups of 6 mice each: one group received the vehicle, another group received Nintedanib orally at 60 mg/kg once daily for 3 weeks (from day 7 to day 28, NINT 7–28), and the third group received Nintedanib orally at the same dose but for 2 weeks (from day 14 to day 28, NINT 14–28).

**Figure 1:**
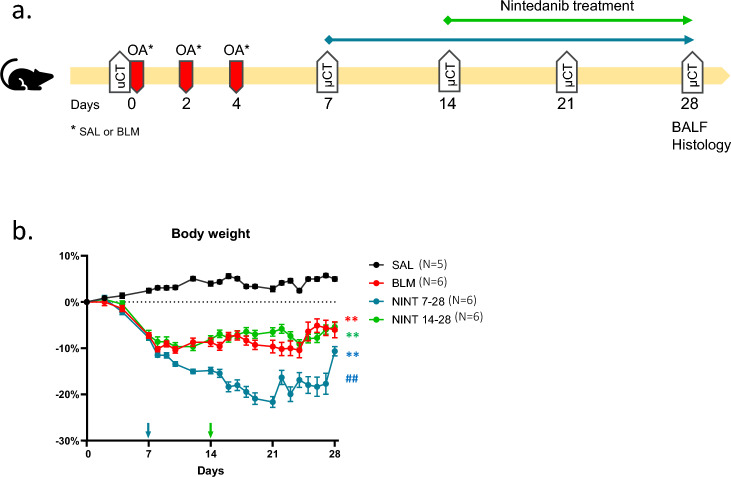
Experimental protocol outline and body weight variation. (**a**) Schematic representation of the experimental protocol illustrating the timing of BLM challenge and treatment with Nintedanib under different treatment regimens. (**b**) Body weight changes from day 0 to the end of the study, expressed as a percentage variation with respect to day 0. Data are shown as mean ± SEM. Two-way ANOVA + Tukey’s multiple comparison. **p < 0.01 versus SAL, **##***p* < 0.01 versus BLM.

A schematic representation of the experimental protocol is shown in Fig. 1a, whilst the composition and sample sizes of the experimental groups are summarized in Table 1.

**Table 1:** Experimental groups.

Group	Treatment	N
SAL	Saline	5
BLM	BLM 6 + 6 + 6 (µg)	6
NINT 14–28	BLM 6 + 6 + 6 (µg) + Nintedanib 60 mg/kg/die p.o	6
NINT 7–28	BLM 6 + 6 + 6 (µg) + Nintedanib 60 mg/kg/die p.o	6

### Micro-computed tomography

#### Micro-CT data acquisition protocol

Mice thoraxes were scanned with a Quantum GX Micro CT instrument (PerkinElmer, Inc. Waltham, MA) at 0 (baseline), 7, 14, 21, and 28 days after anesthesia induction and maintenance with 2% isoflurane. Images were captured in free-breathing mice using the following parameters: X-ray tube current 88 A, X-ray tube voltage 90 kV, and a total angle of 360° during a scan length of 4 min. The detector size was 736 × 588 pixels with a size of 0.2 mm, which were automatically clipped by the reconstruction algorithm to achieve a projection size of 512 × 512 pixels. Binning was not applied. Each animal was positioned supine on the scanner bed, with the chest adjusted to fit within the field of view. To detect the breathing pattern, images were acquired in ‘high speed’ mode, enabling the recording of the gating signal throught a region of interest (ROI) positioned above the diaphragm of the animal. Projections were gathered in ‘list mode’ (projections labeled as “00000”, “00001”, …, “14,687”) over a single continuous gantry rotation. A window indicated the breathing pattern and the position of the projections that would then be used for the reconstruction of the inspiratory (P01) and expiratory (P02) volumes at the end of each acquisition. During the 4 min acquisition time, around 900 projections (both P01 and P02) were automatically sorted and used for the reconstruction of the two datasets, with the option to alter the thresholds to select the more appropriate projections. However, our strictly controlled anesthesia protocol^12^ allowed us to achieve stable breathing rates (100–120 brpm). For each acquisition, two stacks of 512 cross-sectional images were automatically reconstructed into two 3D datasets corresponding to the inspiratory and expiratory breathing phases (i.e., end-inspiration, P01, and end-expiration, P02) with a 50 µm isotropic reconstructed voxel size, using a filtered back-projection algorithm with a Ram-Lak filter. The micro-CT scanner was calibrated monthly, using standard phantoms for noise, homogeneity, low contrast, and resolution^13^.

#### Micro-CT post-processing: Lung segmentation and morphological biomarkers.

The recovered datasets were handled with the DL-based segmentation model^14^. Specifically, for both the end-inspiration and end-expiration phases, the model allowed segmentation of the entire lung as well as of the left and right lungs separately. From the segmented volumes, the tool automatically derived lung volumes (measured in mm^3^) and Mean Lung Attenuation (MLA) densities (measured in Hounsfield Units, HU) together with other biomarkers of interest (see Supplementary Tables 1 and 2). As outlined in Table 2, aeration compartments were also computed using ‘HU preclinical ranges’^15^

**Table 2:** Micro-CT derived morphological biomarkers.

Micro-CT-derived morphological biomarkers			
*Aeration compartments*			
Name	Description	Unit	Formula
%Normo	Percentage of parenchyma that is normo-aerated; reflects the number of no/mild lesions	%	Percent voxels in range [− 860, − 435]
%Hypo	Percentage of parenchyma that is hypo-aerated; reflects the number of moderate lesions	%	Percent voxels in range (− 435, − 121)
%Non	Percentage of parenchyma that is non-aerated; reflects the number of severe lesions	%	Percent voxels in range [− 121, 121]

#### Micro-CT post-processing: lung ventilation maps and functional biomarkers

Lung functional biomarkers were retrieved from micro-CT-derived ventilation maps as previously described^16^. Ventilation maps were obtained by spatially matching inspiratory and expiratory images and calculating the density variation occurring between the two respiratory phases. Image registration was performed using a multi-resolution implementation of the Demons algorithm^17^, following pre-processing of the images with a Laplacian filter to make the registration more sensitive to structures rather than to overall intensity (which varies with lung volume)^16^.

The P01 and the deformed P02 images were segmented by the DL model and subtracted on a voxel‐by-voxel basis to evaluate the inspiratory-expiratory specific gas volume difference (ΔSVg = SVg_P01_—SVg_P02,_ expressed as mL/g). ΔSVg measures the change in the amount of air per voxel relative to tissue mass between the inspiratory and the expiratory phase. SVg is calculated voxel-by-voxel as SVg = specific_volume_lung_—specific_volume_tissue_, with the specific_volume_lung_ (tissue plus gas) measured from micro-CT images as SV_lung_ (ml/g) = 1000/(HU + 1000)^18^ and the specific_volume_tissue_ assumed to be equal to 1/1.065 = 0.939 ml/g^19^. As reported in Table 3, to differentiate between lung regions exhibiting a reduced ΔSVg related to, or unrelated to fibrosis, each voxel in the ventilation maps was categorized into three classes guided by SVg and ∆SVg values^16^.

**Table 3:** Ventilation map derived functional biomarkers.

Ventilation maps derived functional biomarkers			
*Functional ventilation compartments*			
Name	Description	Unit	Formula
%Normal vent	Percentage of normally ventilated lung parenchyma	%	ΔSVg ≥ β
%Low vent	Percentage of lung parenchyma with reduced ventilation, not due to fibrosis	%	ΔSVg < β P01 SVg ≥ $\alpha_{I}$ P02 SVg ≥ $\alpha_{E}$
%Fibrosis	Percentage of parenchyma with reduced ventilation due to fibrosis	%	ΔSVg < β P01 SVg < $\alpha_{I}$ P02 SVg < $\alpha_{E}$

a_I_ and a_E_ represent the 5th percentile values of the inspiratory and expiratory SVg basal mean distributions, respectively; β is the 25th percentile of the ΔSVg basal mean distribution.

### Bronchoalveolar lavage fluid inflammatory cells and cytokine quantification

Mice were humanely euthanized with an overdose of anesthesia, followed by aortic bleeding to ensure euthanasia. Bronchoalveolar lavage fluid (BALF) was then collected by three gentle lung washes using 0.6 mL of a sterile 1 × Hank’s balanced salt solution (HBSS) containing 10 mM ethylenediaminetetraacetic acid (EDTA) and 10 mM 4-(2-hydroxyethyl)-1-piperazineethanesulfonic acid (HEPES) (BALF solution). Following BALF samples centrifugation (300 g for 10 min at 4 °C), the resulting cell pellets were resuspended in 0.2 mL of BALF solution. The resuspended samples were utilized for the quantification of total white blood cells (WBC) and their subpopulations, which was performed with an automated cell counter (Sysmex Dasit XT 1800 J). The supernatants were stored at − 80 °C, and subsequently utilized for the determination of multiple pro-inflammatory cytokines with a Magnetic Luminex Assay (R&D Systems, Minneapolis, MN) following the manufacturer’s instructions.

### Lung histology

Following a gentle infusion of 10% neutral-buffered formalin, lungs were collected, fixed for 24 h and embedded for subsequent analyses. Lung tissue slices, each 5 μm thick, were meticulously sectioned with a Slee Cut 6062 apparatus (Slee Medical, Mainz, Germany). Three sections from each lung sample were stained with Masson’s Trichrome (MT) and scored for fibrosis on a scale ranging from 0 to 8. Fibrotic pathology was also assessed with a modified and independently validated Ashcroft score (AS) system^20–22^. The cumulative score thus obtained was then averaged across all microscopic fields in order to obtain a comprehensive evaluation. To quantitatively analyze pulmonary fibrosis distribution, the Ashcroft scores were classified as: 0 to 3 (mild), 4 (moderate), and ≥ 5 (severe). A NanoZoomer S60 scanner (Hamamatsu Photonics, K.K., Japan) was used to acquire whole-slide images (WSI).

### Statistical analysis

Statistical analyses were performed with the Prism 8 software (GraphPad Software Inc., San Diego, CA, USA); data are given as mean ± SEM. One or two-way analysis of variance (ANOVA) was initially performed, followed by Dunnett or Tukey’s multiple comparison post-hoc tests to enable comparisons among distinct experimental groups. Prior to statistical analysis, normal distribution was assessed with the Shapiro–Wilk test, complemented by visual examination of QQ-plots. Sample size determination was performed with A-priori Power Analysis (GPower Version 3.1.2), using the Ashcroft Score as endpoint. Finally, the correlation between micro-CT and histological readouts was assessed by calculating Spearman correlation coefficients. In all tests and experiments, the statistical significance threshold was set at a *p* value < 0.05.

## Results

The overall experimental design, including the BLM and NINT dosing schedule and the timing of micro-CT imaging is outlined in Fig. 1a.

### Body weight

Body weight of the mice was recorded throughout the study. As shown in Fig. 1b, starting from day 7 all BLM-treated animals featured a significant weight loss compared to the SAL control group (*p* < 0.01). Weight loss progressed in the group whose NINT treatment started on day 7 and, despite a slight recovery at late time-points, it persisted until the end of the study. Such a weight loss progression was not observed in the group that started NINT treatment on day 14, which displayed a weight loss trend nearly identical to that of the BLM-only group.

### Micro-CT-derived lung morphological biomarkers

**Figure 2:**
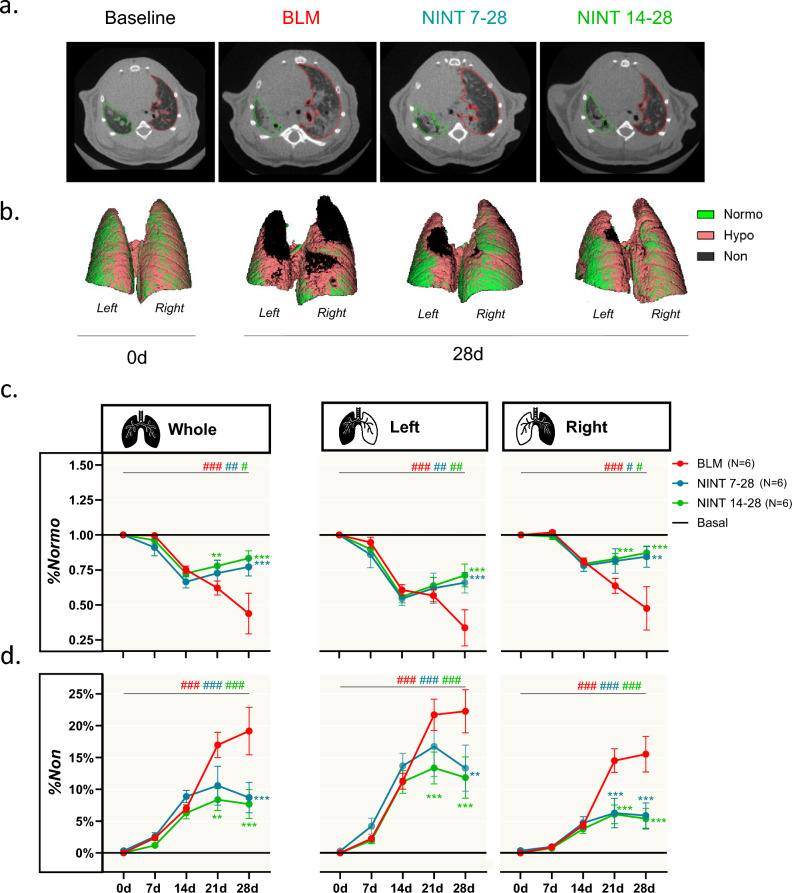
Whole-, left- and right-lung aeration compartment. (**a**) Represntative 2D axial slices with corresponding segmentation masks (left lung in *green*, right lung in *red*) derived at baseline and at the terminal endpoint (28d). (**b**) 3D representation of the aeration compartments visualized by micro-CT in a representative set of BLM-only or NINT-treated mice as indicated at the end of the study (28d); animals imaged a time zero, before bleomycin administration (0d) served as controls. The lung was normally aerated at day 0, whereas a large non-aerated volume located in the upper portion of the lung became apparent in the BLM animal at day 28. Much smaller non-aerated portions, predominantly located in the left lung, are present in NINT 7–28 and NINT 14–28 mice. (**c**) Longitudinal quantification of %Normo expressed as fold-change with respect to the basal condition, represented by the black line. (**d**) Same as in (**c**) for %Non. BLM (*red*), NINT 7–28 (*blue*), and NINT 14–28 (*green*) data are given as mean ± SEM. All data were derived from end-expiratory images. Statistical significance of the longitudinal changes revealed by micro-CT-derived parameters in the different groups relative to the basal condition was assessed by two-way ANOVA followed by Šidák post-hoc test (#*p* < 0.05; ##p < 0.01; ### p < 0.001 0d vs 28d). Statistical significance of the differences between the NINT and the BLM groups was calculated by two-way ANOVA followed by Dunnett’s t post-hoc test (*p < 0.05; **p < 0.01; ***p < 0.001 vs. BLM group).

Figure 2a shows axial slices depicting the relative segmentation masks of representative animals at day 0 (baseline) and at day 28. A corresponding panel illustrating the coronal slices for these animals at each of the investigated time-points is provided in Supplementary Fig. 1.

The renderings derived from the segmentation masks depicted in Fig. 2a are represented in Fig. 2b, and color-coded according to the aeration compartments at end-expiration. The basal state was characterized by a well-aerated lung parenchyma (%Normo equal to 75%, *green* areas), which, albeit to different extents, diminished in all groups by the end of the experiment (day 28). In the BLM-only mouse, extensive non-aerated regions dominated the apexes of the lung (%Non equal to 23%, *black* areas). An intermediate situation was observed in both NINT 7–28 and NINT 14–28 animals, in which the right lungs were mainly characterized by normally aerated tissue (%Normo above 70% in both groups), whereas a well-detectable amount of non-aerated tissue was present in the left lungs (%Non ~ 10% in both groups).

These trends were confirmed by the longitudinal analysis conducted on each group. As expected, the SAL controls displayed stable metrics at all time-points (Supplementary Fig. 2), whereas significant changes were observed in all the other groups. As shown in Fig. 2c, which reports the extent of normally aerated tissue (%Normo) of BLM, NINT 7–28, and NINT 14–28 animals, expressed as fold change with respect to their basal levels, %Normo decreased from day 0 to day 28 in all groups, both in the whole lung and in individual lobes. In the NINT 14–28 group, already at day 21 (i.e., after 1 week of treatment), %Normo was significantly higher than in the BLM-only group in both the whole and the right lung (whole lungs: *p* < 0.01; right lung: *p* < 0.001). A significantly higher level of %Normo compared to the BLM group was observed in the left lobe not earlier than day 28 (*p* < 0.001). Interestingly, the NINT 7–28 group displayed significantly higher levels of %Normo compared to BLM only on day 28, i.e., after 3 weeks of treatment (whole and left lung: *p* < 0.001; right lung: *p* < 0.01). Likewise, the extent of non-aerated tissue (%Non) increased in all groups from day 0 to day 28 (*p* < 0.001), especially in the left lung (Fig. 2d). Significant differences, i.e., reduced levels of %Non with respect to the BLM group, were observed starting from day 21 in the NINT 14–28 group in the whole (*p* < 0.01), left and right lung (*p* < 0.001). In the NINT 7–28, these differences were limited to the right lung (*p* < 0.001). On day 28, both NINT groups displayed significantly lower levels of %Non compared to the BLM group, in the whole (*p* < 0.001), left (*p* < 0.01), and right lungs (*p* < 0.001). Additional morphological data were retrieved from micro-CT images (Supplementary Fig. 3). Briefly, these pointed to an initial increase in whole lung volumes due to inflammation across all groups, which subsequently decreased as left lung volumes returned to baseline and right lung volumes were stabilized (Supplementary Fig. 3a). A marked decline in %Gas_P02_ was observed in all groups on day 14, with a subsequent recovery by day 28 only occurring in the NINT groups (Supplementary Fig. 3b). It should also be noted that the elevated lung volumes observed on day 7 corresponded to increased tissue volumes (Tissue), which remained elevated in the right lung even at later time points (Supplementary Fig. 3c). Significant differences in these biomarkers between the BLM and the NINT groups began to manifest on day 21 and were appreciably more evident in the NINT 14–28 group. Tidal Volume (TV) analysis (Supplementary Fig. 3d) highlighted the inability of whole lung assessments alone to capture the compensatory dynamics occurring after triple BLM administration. In fact, the apparent lack of any detectable change in TV at the whole lung level was accompanied by a markedly impaired TV in the left lobe and an opposite compensatory trend in the right lobe, with a TV exceeding or matching baseline levels. Regardless of these lobe-specific variations, no significant differences between the BLM and the NINT groups at the level of TV could be detected.

### Micro-CT-derived lung ventilation maps and functional biomarkers

**Figure 3:**
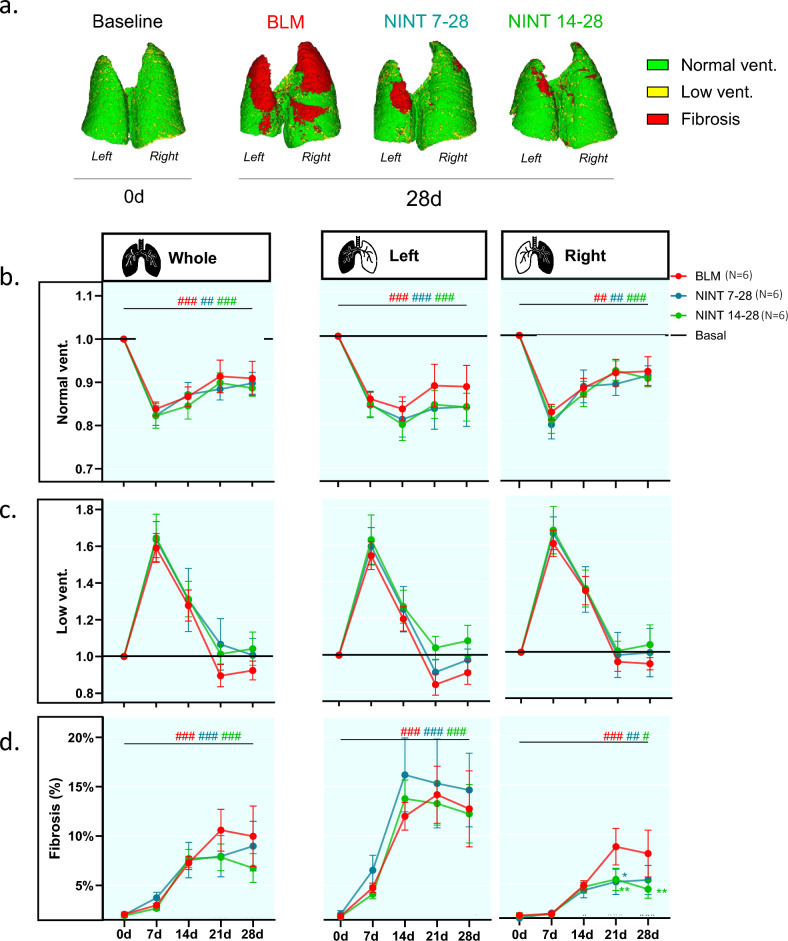
Functional ventilation compartments in the whole-, left-, and right-lung. (**a**) 3D images of the functional ventilation compartments of representative mice before BLM administration (0d) and at the end of the study (28d). On day 0, the lungs are almost entirely classified as normally ventilated (*green*). On day 28 lung functionality was impaired in all mice due to fibrosis (red). (**b**) Longitudinally evaluated normal ventilation (%Normal vent.) expressed as mean fold-change with respect to the basal condition, represented by the black line. (**c**) Same as in (**b**) for low ventilation (%Low vent.) (**d**) Progression of fibrosis expressed as mean percentage values. BLM (*red*), NINT 7–28 (*blue*), and NINT 14–28 (*green*) data are given as mean ± SEM. Statistical significance of the longitudinal changes with respect to the basal condition in micro-CT-derived parameters among the various groups was assessed by two-way ANOVA followed by Šidák post-hoc test (#*p* < 0.05; ##*p* < 0.01; ###*p* < 0.001 0d vs. 28d). Statistical significance of the differences between groups was calculated by two-way ANOVA followed by Dunnett’s t post-hoc test (*p < 0.05; **p < 0.01; ***p < 0.001 vs. BLM group).

The ventilation compartments derived from the same mice of Fig. 2a, b are displayed in Fig. 3a. As expected, the basal lung was characterized by overall normal ventilation (Normal vent ~ 80%; *green* areas), with only a few low ventilation regions (Low vent ~ 20%; *yellow*). On day 28, the BLM animal featured extensive fibrosis (Fibrosis ~ 20%; *red*) predominantly localized to the lung apexes of both the left and the right lobes. In contrast, NINT-treated animals displayed a normally ventilated lung parenchyma, with only a few fibrotic areas (Normal vent ~ 75% and Fibrosis ~ 10% in both NINT groups), mainly located in the apical part of the left lungs.

Figure 3b, c show the longitudinal variation in the extent of normally ventilated (%Normal vent.) and low ventilated lung regions (%Low vent.) expressed as fold-change with respect to their basal levels. Similar to the aeration compartments, the BLM group consistently displayed a lower %Normal vent. compared to its baseline level at all time-points, both in the whole lung and in the left lobe. In the right lobe, following a marked drop on day 7, %Normal vent. levels partially recovered on days 21 and 28. No statistically significant differences in %Normal vent. and %Low vent. were detected between the BLM and the NINT groups. Interestingly, a clear trend was apparent in all groups and lung regions for %Low vent. (Fig. 3c), which increased by more than 1.5-fold on day 7, then declined to 1.2-fold on day 14, and approached baseline levels at later time-points. As expected, fibrosis, which is given as a raw percentage in Fig. 3d since it was close to 0% at baseline, significantly increased from day 0 to day 28 in the whole, right, and left lungs (*p* < 0.01) of BLM mice. The increase in %Fibrosis, however, was not homogeneously distributed across the lung parenchyma. The left lung, in particular, exhibited the largest increase in %Fibrosis, with values exceeding 10% in all groups on days 14, 21, and 28. It should also be noted that, although not significantly, %Fibrosis in the left lung of the NINT 7–28 group appeared to be slightly higher than that of the BLM animals, especially on days 14, 21, and 28. Differences in %Fibrosis among the BLM and the NINT groups were limited to the right lung, with a significant reduction detected in both the NINT 7–28 (*p* < 0.05) and NINT 14- 28 (*p* < 0.01) groups at day 21, and a further reduction at day 28, that only reached significance (*p* < 0.01) in the NINT 14–28 group.

### Lung histology

Representative images of whole lung and left lung sections (20 × magnification) from all experimental groups, stained with hematoxylin and eosin (H&E) and Masson’s Trichrome (MT), are shown in Supplementary Fig. 4a (H&E) and b (MT);

As expected, histological examinationin BLM-treated mice, revealed patchy fibrotic lesions characterized by fibroproliferative foci (Supplementary Fig. 4b, *red arrows*) with varying degrees of confluence at 28 days. These were less pronounced in NINT 7–28 and NINT 14–28 animals, and completely absent in SAL controls.

**Figure 4:**
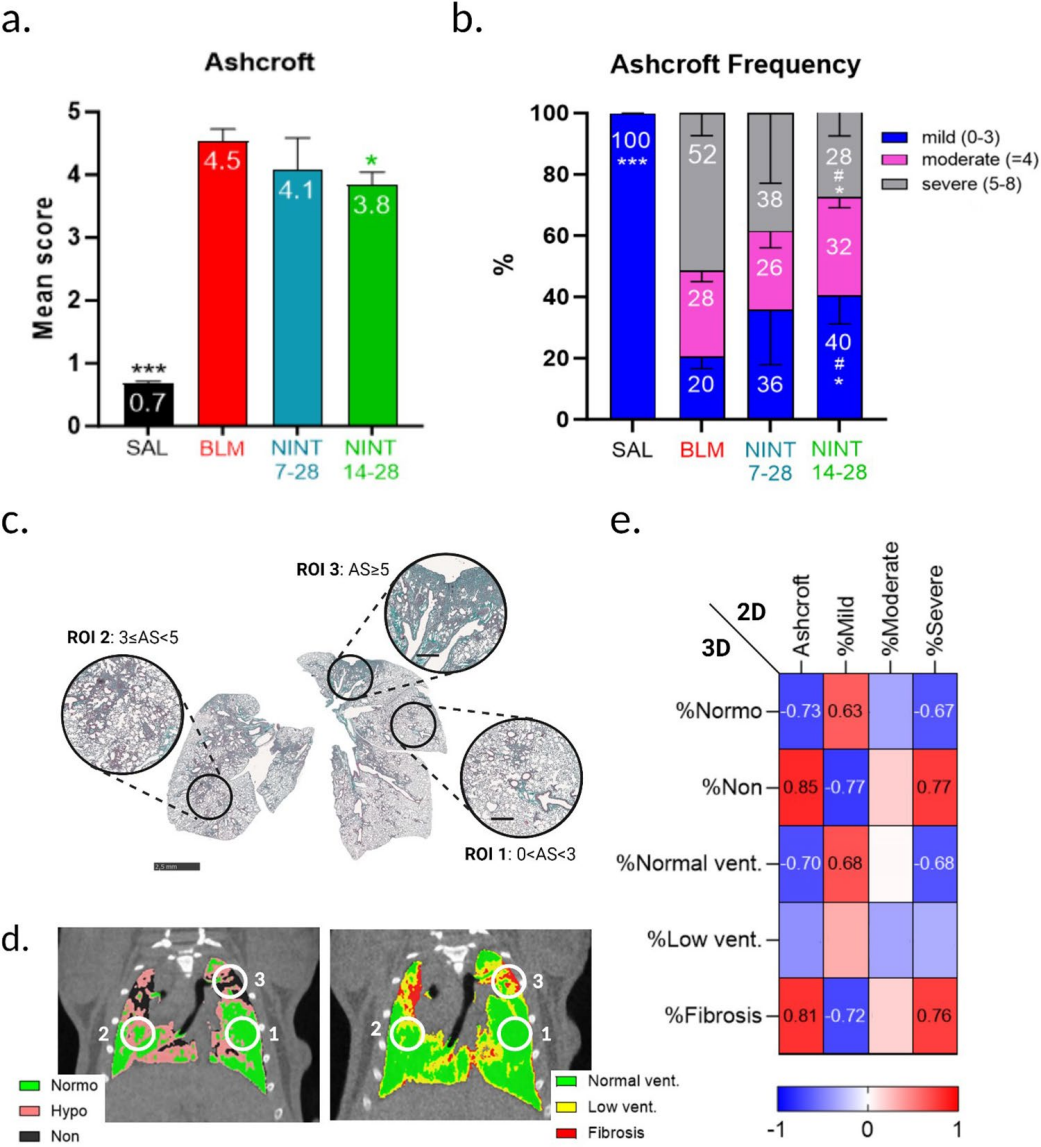
Histological assessment. Fibrotic lesions accumulation in the lungs at day 28 was revealed in the indicated groups by (**a**) mean Ashcroft Score and (**b**) Ashcroft Frequency distribution of the Ashcroft classes (mild, moderate, and severe). Data were expressed as mean ± SEM. For Ashcroft Score the one-way ANOVA followed by Dunnett’s multiple comparison was used to evaluate the statistical significance of the differences (*p < 0.05; **p < 0.01; ***p < 0.001, vs. BLM group), whereas for Ashcroft Frequency distribution was employed the two-way ANOVA followed by Dunnett’s t post-hoc test (*p < 0.05; **p < 0.01; ***p < 0.001 vs. BLM group). Student’s t-test analysis was repeated for each AS class frequency to evaluate the statistical significance between NINT 7–28 and NINT 14–28 (#*p* < 0.05; ##*p* < 0.01). (**c**) Representative whole slide histology (bar: 2.5 mm) of a randomly selected mouse of the BLM group stained with Masson’s Trichrome (MT). Circles indicate regions of interest (ROIs) reported alongside with higher magnification (bar: 500 µm) and displaying different degrees of fibrotic lesions (ROI_1_: mild, ROI_2_: moderate, and ROI_3_: severe). (**d**) Representative CT-derived coronal slices with segmentation masks of the same randomly selected mouse of the BLM group. On the left, end-expiratory phase, voxels inside the lungs were classified into aeration compartments. On the right, end-inspiratory phase, voxels inside the lungs were classified into functional ventilation compartments. White circles represent the location in CT of the ROIs identified in histology. ROI_1_: mild AS, 97% Normo + 3% Hypo aerated tissue; 100% Normal ventilation; ROI_2_: moderate AS, 10%Non + 33% Hypo + 67% Normo aerated tissue, 70% Normal. + 30% Low. ventilated areas; ROI_3_: 45% Non + 44% Hypo + 11% Normo aerated tissue, 50% Non vent + 30% Low + 10% Normal ventilated areas. (**e**) Heat-map representation of Spearman correlation coefficients between micro-CT-derived biomarkers and histological parameters in the whole lungs; no-significant correlations (*p* value > 0.05) were not displayed.

As shown in Fig. 4a, a significant increase in the Ashcroft score was observed in the BLM group compared to the SAL group (*p* < 0.001). NINT 14–28 treatment markedly reduced the Ashcroft score (*p* < 0.05 vs. BLM), whereas a modest, statistically non-significant reduction was produced by the NINT 7–28 treatment. No statistically significant differences were measured between Ashcroft scores mean values of the NINT 7–28 and of the NINT 14–28 groups. The frequency distribution of the Ashcroft score (Fig. 4b) indicates that BLM treatment significantly elevated the levels of moderate and severe fibrosis compared to the SAL group (*p* < 0.001). The NINT 14–28 group underwent a significant reduction in severe grades compared to the BLM group (*p* < 0.05), with a marked increase in mild grades (*p* < 0.05). A similar trend, i.e., an increase in moderate grades and a drop in severe grades, was observed in the NINT 7–28 group, which, however, did not result in statistically significant differences compared to the BLM mice. Nevertheless, a significant difference between NINT 7–28 and NINT 14–28 was measured in the extent of fibrotic lesions classified as mild or severe.

2-D histological slices from a representative BLM-treated mouse are shown in Fig. 4c. Three distinct regions of interest (ROIs) were identified, each corresponding to a different histological severity grade (mild, moderate, and severe). Figure 4d highlights the corresponding ROIs on a representative coronal slice derived from micro-CT imaging.

The strong correlation between micro-CT and histology is confirmed by the comparison of all histological 2-D parameters (mean AS, mild, moderate, and severe grades) with the 3-D micro-CT-derived aeration and functional compartments measured at day 28 for all mice (Fig. 4e).

Despite histology being conducted on a single coronal 2-D slide and micro-CT data quantification being performed on 3-D lung volumes, the two assessments showed a high level of correlation. However, the %moderate parameter in histology, and %Low vent. in the micro-CT data, exhibited lower correlation coefficients with respect to the other investigated parameters.

### BALF cells count

**Figure 5:**
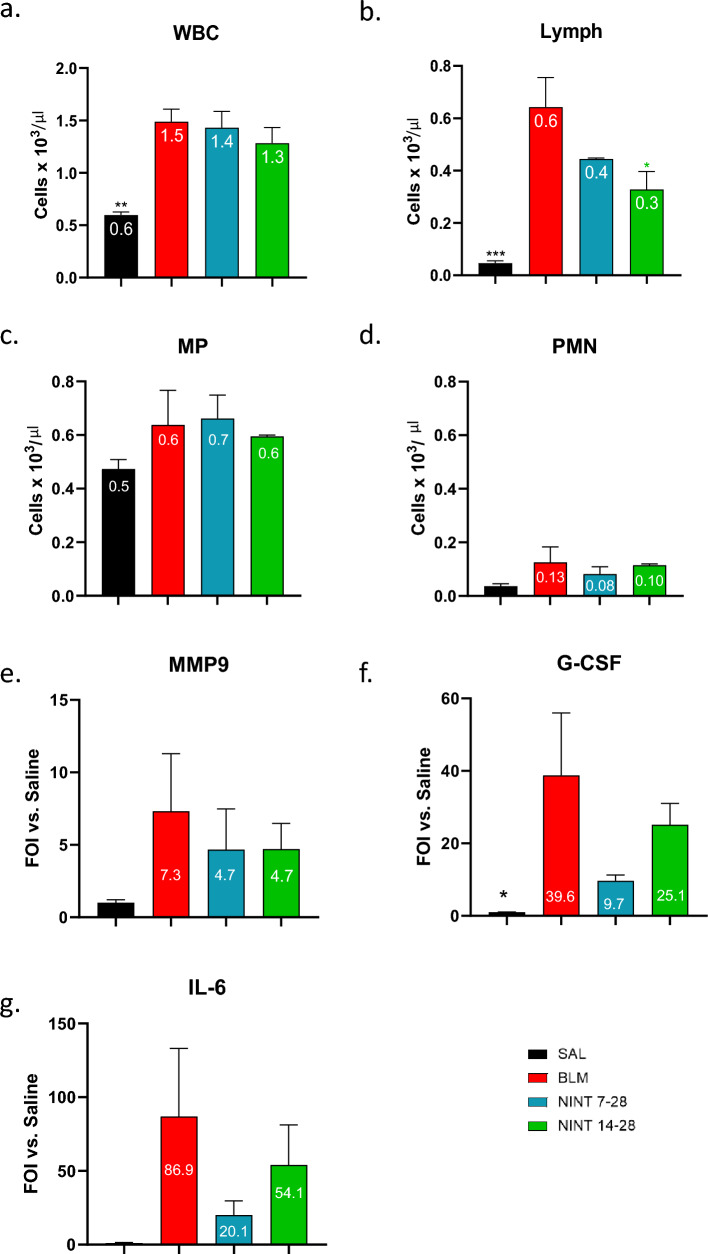
BALF cell populations, cytokines, and MMP-9 levels. (**a**) Total white blood cell levels (WBC), (**b**) Lymphocyte (Lymph), (**c**) Macrophages (MP), and (**d**) polymorphonuclear (PMN) granulocytes counts in the BALF of the SAL, BLM, NINT 7–28 and NINT 14–28 groups at day 28. (**e**) MMP-9, (**f**) G-CSF and (**g**) IL-6 levels were measured in the BALF supernatants of the indicated groups at day 28 and given as fold of induction (FOI) compared to the SAL control group; data were expressed as mean ± SEM. One-way ANOVA followed by Dunnett’s Multiple Comparison was used to evaluate the statistical significance of the differences (*p < 0.05; **p < 0.01; ***p < 0.001, vs. BLM group).

Cell count analysis of BALF samples performed at day 28 on all animals revealed a significant increase (*p* < 0.01) in total white blood cells (WBC) in the BLM group compared to the SAL controls, with an appreciable but not statistically significant reduction in the NINT groups (Fig. 5a). Lymphocytes were particularly elevated in the BLM compared to the SAL mice (*p* < 0.001), and only the NINT 14–28 treatment resulted in a significant reduction of lymphocyte levels (*p* < 0.05, Fig. 5b).

Macrophages (MP) levels, instead, were increased in the BLM group compared to SAL mice, even if not significantly, and were thus not affected by neither of the NINT treatment regimens (Fig. 5c). Despite a slight increase in the BLM mice, polymorphonuclaear (PMN) cells levels were uniformly low across all experimental groups and no significant differences were observed (Fig. 5d).

We also determined the levels of soluble inflammatory and fibrotic mediators in the BALF supernatants. This analysis revealed increased levels of the MMP-9 metalloprotease (Fig. 5e) in the BLM compared to the SAL mice, which, however, were only slightly and non-significantly affected by either NINT treatment. Similarly, G-CSF levels were significantly increased in the BLM group (*p* < 0.05), yet were only modestly, and non-significantly modified by either NINT treatment (Fig. 5f). An even flatter response was displayed by the IL-6 cytokine, whose increase in the BLM-only group and lowering by either NINT treatment failed to reach statistical significance (Fig. 5g). In summary, a significant increase in total WBC and lymphocyte levels is induced by BLM at day 28, which is significantly alleviated by the NINT 14–28 but not the NINT 7–28 treatment.

## Discussion

In response to the limitations of the double BLM-induced lung fibrosis model, which include the spontaneous resolution of fibrosis after 21 days and an initial acute inflammatory phase that takes up a significant portion of the overall experimental window, we recently proposed an alternative mouse model of pulmonary fibrosis. This alternative model involves a triple BLM administration that leads to a fibrotic condition lasting for at least 28 days^10^ In addition to its longer-lasting pro-fibrotic effect, the triple BLM administration further separates the initial inflammatory response elicited by BLM from the pure fibrotic phase. This expanded duration thus extends the potentially useful time-window for the testing of new candidate antifibrotic drugs and may allow to avoid potential confounding effects associated with overlapping inflammatory and fibrotic responses. In this work, we tested both potential advantages by investigating the optimal time-window for treatment with Nintedanib, a widely used reference therapeutic compound in the BLM model.

*In-vivo* longitudinal morpho-functional data derived from micro-CT imaging as well as the results of *ex-vivo* histo-morphometric measurements indicated that the most effective time-window for Nintedanib treatment is between days 14 and 28. In fact, at different time-points and in different lung samples, NINT 14–28 significantly outperformed the NINT 7–28 treatment in 14 out of 31 measured outcomes, including morphological and other micro-CT-derived biomarkers (Fig. 2, and Supplementary Fig. 3), Ashcroft score and lymphocyte counts (Figs. 4a, 5b).

Additionally, earlier administration of Nintedanib (7–28) did not result in any appreciable improvement in fibrosis during the first week of treatment (day 7 to 14). Instead, it induced an overall increase in animal stress, as evidenced by the rapid weight loss observed in the NINT 7–28 group during the initial week, with a lack of significant recovery thereafter. However, by day 28, a moderate recovery was observed.

This strongly suggests that initiating NINT treatment on day 7, when the inflammatory phase is at its peak^10^, far from improving the therapeutic outcome, could worsen the distress caused by BLM administration, making this treatment regimen less effective and poorly tolerated.

In accordance with previous studies^23^, our results suggest that side-effects and poor animal health may curtail the therapeutic response to the bleomycin challenge. Moreover, regardless of the treatment regimen applied, no net improvement in body weight was observed in NINT-treated animals. This resembles the situation of Nintedanib-treated IPF patients, who, despite a reduced progression of fibrosis markers, withstand quite a few side-effects, without any significant improvement in the overall quality of life^3^.

Micro-CT was instrumental to a reliable longitudinal assessment of fibrosis progression and response to treatment. It allowed to automatically derive both lung morphology-related (e.g., the early increase in lung volume reflecting inflammation and the later increase in lung density (%Non) due to collagen deposition and fibrosis progression) and region-specific functional biomarkers (e.g., ventilation). Indeed, most (12 out of 14) of the biomarkers differentiating the NINT 7–28 and 14–28 treatments were derived from micro-CT analysis. Interestingly, none of these differentiating biomarkers involved ventilation, which remained essentially the same in all groups, except for a slight region-specific improvement restricted to the right lung. Overall, longitudinal morphological data analysis revealed that Nintedanib can hinder fibrosis progression, as revealed by the increase of Non-aerated and decrease in Normo-aerated areas, as compared to the control BLM group. However, it has proven to be ineffective in reversing the pathology, thus incapable of producing any actual improvement in the well-being of mice as witnessed by the body weight decrease. Since the functional parameters depend on the presence of fibrotic lesions and reflect the animals’ quality of life, we can speculate that these are the reasons why Nintedanib appeared to be more targeted at reducing pulmonary fibrosis than improving ventilation. Moreover, in the left lung, which experienced a more pronounced ventilation impairment, Nintedanib treatment not only proved ineffective in restoring lung functionality but, when initiated on day 7, it tended to exacerbate the lung function impairment caused by BLM administration.

Similar to the double BLM model^11,16^, the triple BLM administration caused a time-dependent increase in severe fibrotic lesions primarily localized to the lung apexes, with a marked preference for the left lobe. These lesions appeared as patchy changes in lung parenchyma density that were accompanied by an uneven reduction in ventilation, also primarily affecting the left lung. This progressive functional impairment was initially driven by the rapid expansion of regions of low ventilation on day 7, followed by their decline and simultaneous increase in fibrotic areas on day 14. Regions of low ventilation returned to baseline levels by day 21, but fibrotic areas continued to accumulate, particularly in the left lung, and remained essentially stable throughout the study.

These results, on one hand, corroborate the observation that low ventilation may serve as a valuable marker of inflammation^16^. On the other hand, they provide further support to the notion that Nintedanib administration during the early phase of the BLM model (either double or triple), i.e., when inflammation predominates over the fibrogenic response, may confound the interpretation of its anti-fibrotic effects^24^. Indeed, no differences were observed between BLM and NINT animals within the time window from day 7 to day 14.

In contrast with the high variability of histo-morphometric parameters and hydroxyproline lung content previously reported in the BLM model after day 21^25^, the fibrotic alterations revealed in the triple-BLM model by micro-CT, both morphologically and functionally, are consistent with stable fibrosis lasting till the very end of the experimental time-window utilized in the present study (days 21–28). Regardless of the treatment regimen (days 7–28 or 14–28), the antifibrotic effect of Nintedanib was only detectable within aeration compartments, particularly in the right lung, from day 21. This suggests that the anti-fibrotic potential of Nintedanib (and, likely, of other antifibrotic drug candidates) can be assessed after just 1 week of treatment only if the animals are dosed after the inflammatory phase has subsided.

In line with previous findings^11^, the triple BLM model shows clear signs of compensatory responses. This was highlighted by the above-baseline tidal volumes (TV) values measured in the right lung of all groups, which strongly hint at compensation for a reduced TV in the left lung (see Supplementary Fig. 3d). Also, the patchy distribution of fibrotic lesions, which based on the results of our semi-quantitative analysis were found to be mainly localized in the lung apexes, points to the caudal lung region as the main site of such compensatory response. To fully validate this phenomenon, however, the TV should be further investigated by quantitative regional and longitudinal measurements capable of differentiating between the contribution to ventilation of apical and caudal lung regions.

Histological analyses confirmed the presence of fibrotic alterations in BLM-treated animals. The Ashcroft score mean values and the distribution of fibrosis grades were significantly modulated only by Nintedanib administrated between days 14 and 28. However, statistically significant differences between the two treatment regimens were found only in the amount of mild and severe fibrotic lesions (*p* < 0.05). The strong correlation observed between imaging and histological results offers a notable advantage in terms of the time needed to assess treatment response. Typically, histological data take several weeks to become accessible to investigators. However, having a faster readout through in-vivo imaging that can reliably predict histological outcomes would facilitate decision-making in drug discovery projects.

The findings provided by micro-CT and histology were further corroborated by BALF analyses revealed significantly increased white blood cell and lymphocyte levels in the BLM group. Again, these effects, especially the increased lymphocyte levels, were more strongly alleviated by the NINT 14–28 than by the NINT 7–28 treatment. Similar increases in the BLM group compared to the SAL controls were observed for other BALF constituents such as macrophages, the MMP-9 metalloprotease, IL-6 and G-CSF. However, despite variable trends toward a Nintedanib-mediated reduction -with a somewhat more pronounced effect of the NINT 7–28 regimen^10,26^ none of the above biomarkers was significantly modified by either NINT treatment. In contrast, no significant variation between the SAL, BLM, and NINT groups was observed for PMN neutrophils, confirming that the inflammatory process had been strongly attenuated by day 28.

In conclusion, the present study, which documents the improved features of the triple BLM model, highlights the importance of a proper time-window for preclinical antifibrotic treatments to disclose the true potential of new candidate drugs. The NINT 14–28 treatment proved to be the most effective against fibrosis, as measured by both micro-CT and histology. In contrast, the extended NINT 7–28 regimen demonstrated reduced effectiveness on purely fibrotic biomarkers, despite exhibiting a more profound modulation of IL-6 and GCSF in the BALF compared to NINT 14–28.

We also showed that far from improving antifibrotic efficacy, a longer exposure to Nintedanib exacerbated its stressful side-effects, thus indicating that starting NINT treatment when the inflammatory phase is not completely resolved may lead to a delayed and less effective antifibrotic response. This underscores the importance of commencing NINT treatment only when the inflammatory phase is fully resolved.

From a methodological point of view, we have integrated, for the first time, structural data obtained during the end-expiratory phase with the functional ventilation compartments derived from co-recording of both end-inspiratory and end-expiratory phases. This combined approach, although not readily available to all laboratories, considers the intrinsic structural and functional complexity of the lungs and is thus crucial for a reliable evaluation of the efficacy of novel lung-targeting drug candidates. In the present study, for example, it allowed to identify the specific issues associated with an approved IPF drug that improves lung density and overall structure but fails to significantly restore functionality. Therefore, we believe that the simultaneous structure–function analysis enabled by longitudinal micro-CT, especially when coupled with a more robust experimental set-up such as the one allowed by the triple BLM model with delayed treatment, can boost the accuracy and reliability of preclinical readouts thus enhancing their translational relevance.

### Supplementary Information

Supplementary Figures.

Supplementary Tables.

## Data Availability

All data generated or analyzed during this study are included in this published article and its supplementary information files.

## Abbreviations

IPF: Idiopathic Pulmonary Fibrosis
BLM: Bleomycin
NINT: Nintedanib
CT: Computed tomography
micro-CT: Micro computed tomography
BALF: Bronchoalveolar lavage fluid
FVC: Forced Vital Capacity
LLMMPF: Longer-lasting mouse model of pulmonary fibrosis
VAS: Visual Analogue Scale
OA: Oropharyngeal aspiration
SAL: Saline
P01: Inspiratory
P02: Expiratory
DL: Deep learning
MLA: Mean Lung Attenuation
HU: Hounsfield Units
ΔSVg: Specific gas volume difference
SVg: Specific gas volume
HBSS: Hank’s balanced salt solution
EDTA: Ethylenediaminetetraacetic acid
WBC: White blood cells
PMN: Polymorphonuclear
MT: Masson’s Trichrome
AS: Ashcroft score
WSI: Whole-slide images
ANOVA: Analysis of variance
