# Supplementary material for: Micro-CT-assisted identification of the optimal time-window for antifibrotic treatment in a bleomycin mouse model of long-lasting pulmonary fibrosis
Source: Sci Rep. 2024 Jun 26;14:14792. doi: 10.1038/s41598-024-65030-3 (PMC11208517; doi:10.1038/s41598-024-65030-3)
Supplement: Supplementary file 1 — Supplementary Figures. [file 41598_2024_65030_MOESM1_ESM.docx]

**Micro-CT-assisted identification of the optimal time-window for antifibrotic treatment in a bleomycin mouse model of long-lasting pulmonary fibrosis**

Martina Buccardi^1,2^**^#^**, Andrea Grandi^2^**^#^**, Erica Ferrini^2,3^, Davide Buseghin^4, 5^, Gino Villetti^2^, Maurizio Civelli^2^, Nicola Sverzellati^6^, Andrea Aliverti^4^, Francesca Pennati^4^ and Franco Fabio Stellari^2*^

**^1^ University of Parma, Department of Mathematical, Physical and Computer Sciences, Parma, Italy**

**^2^ Experimental Pharmacology & Translational Science Department, Chiesi Farmaceutici S.P.A, 43122, Parma, Italy**

**^3^ University of Parma, Department of Veterinary Science, Parma, Italy**

**^4^ Dipartimento di Elettronica, Informazione e Bioingegneria, Politecnico di Milano, Milano, Italy**

**^5^ ANTHEM (AdvaNced Technologies for Human-centrEd Medicine), Spoke 3, Italy**

^6^ **University of Parma, Department of Medicine and Surgery, Parma, Italy.**

**^#^**Martina Buccardi and Andrea Grandi contributed equally

^*^**Corresponding author:** Franco Fabio Stellari ([fb.stellari@chiesi.com](mailto:fb.stellari@chiesi.com)), ORCID: 0000-0002-8855-7297

**
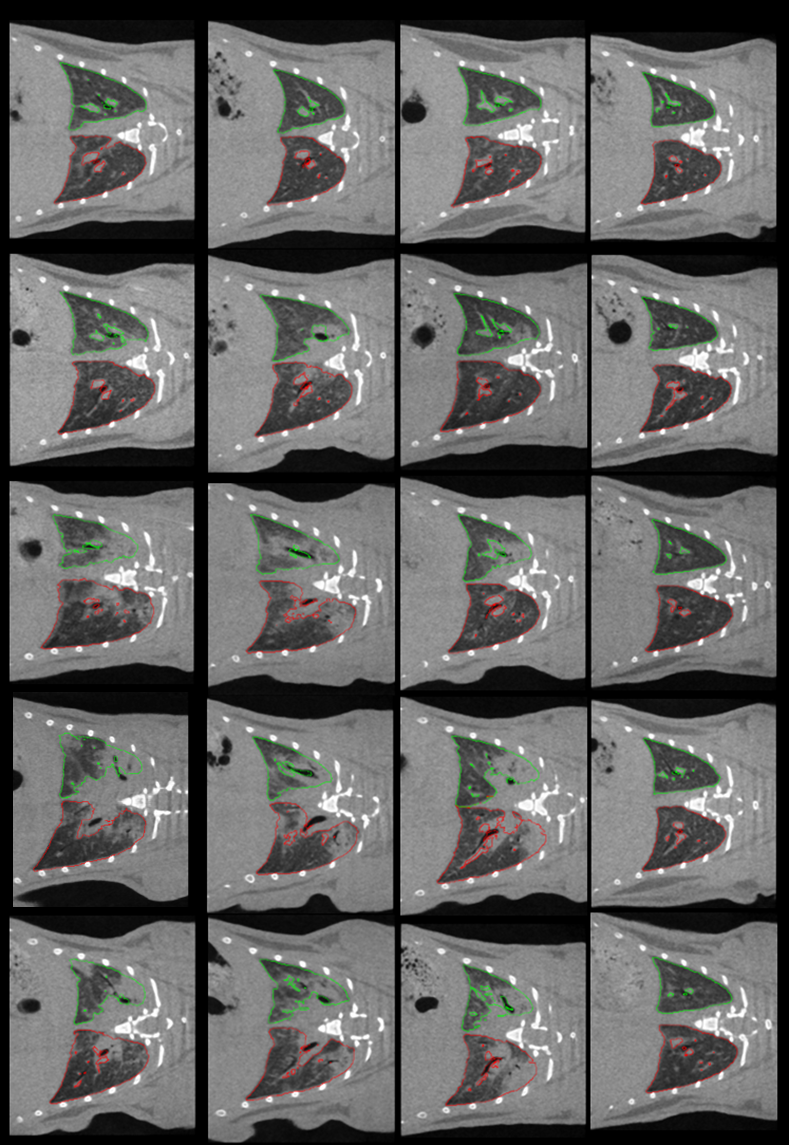

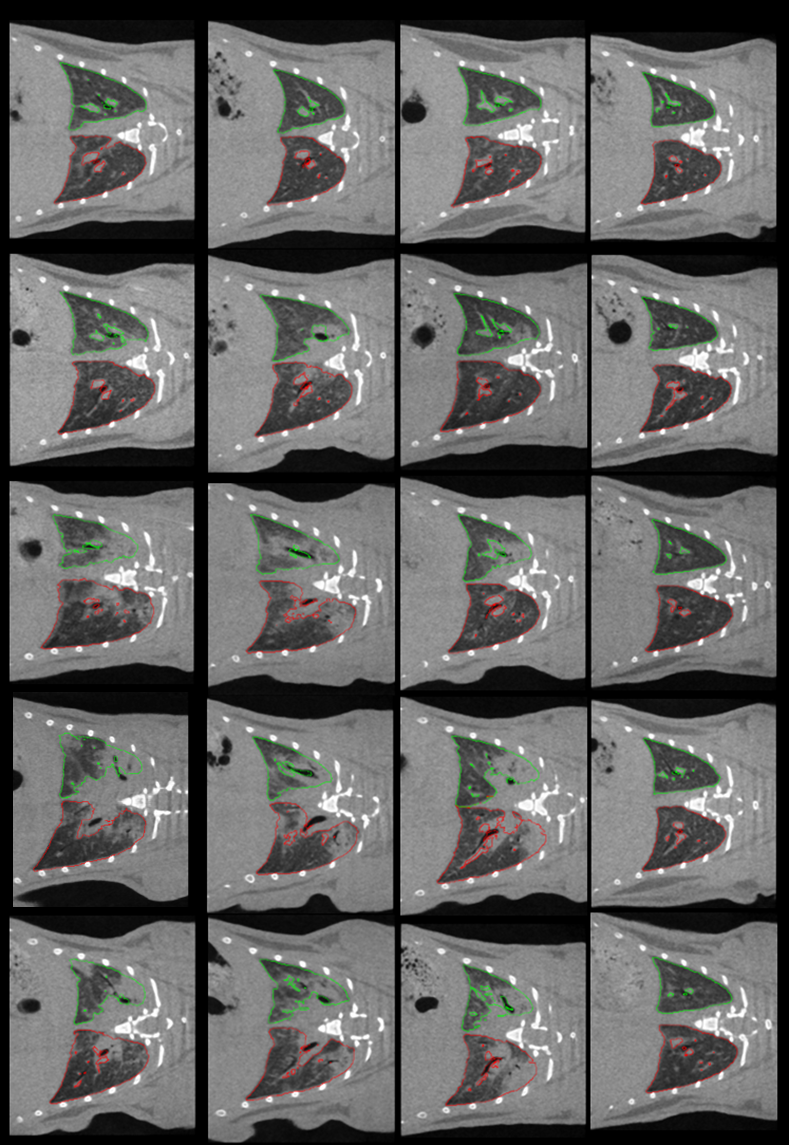

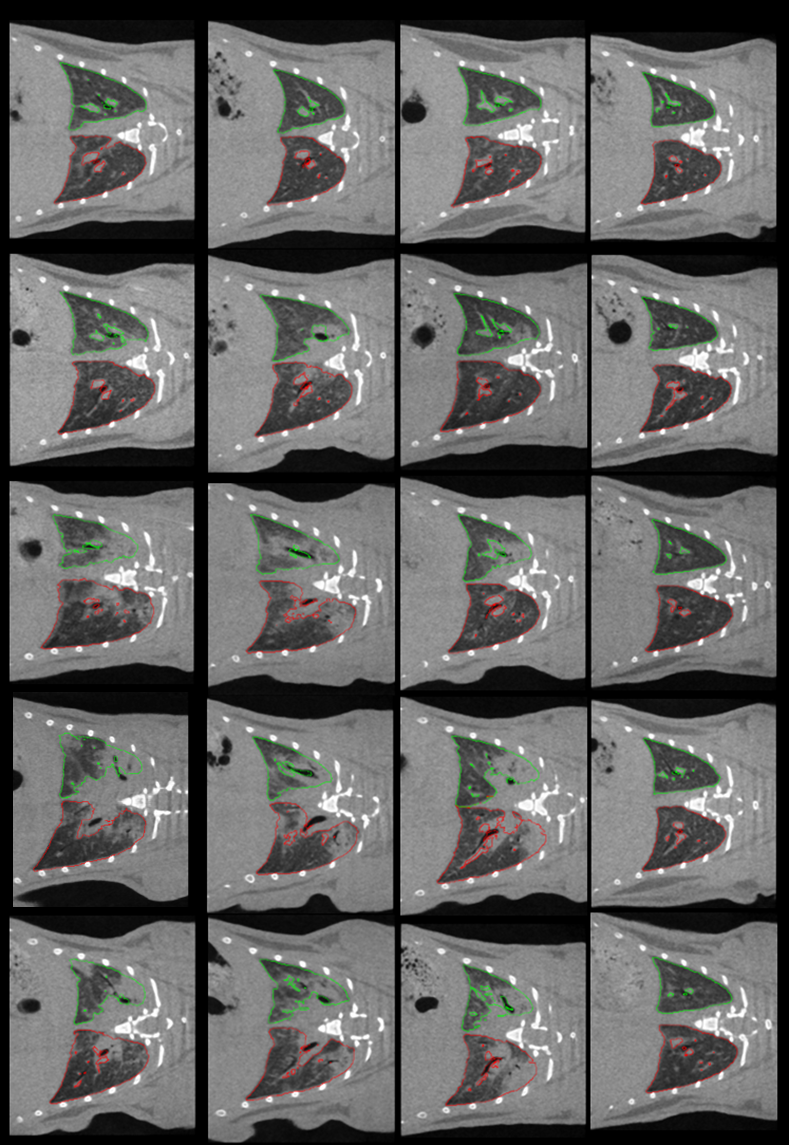

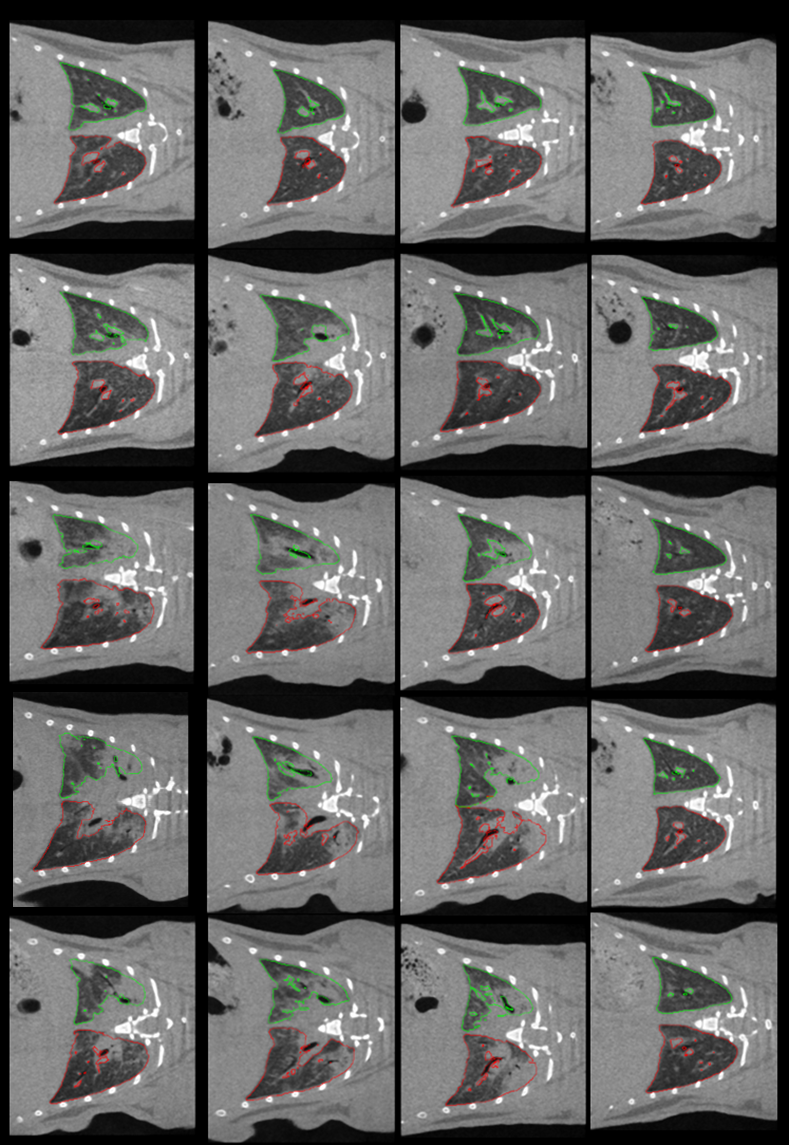
**

28d 21d 14d 7d baseline

SAL BLM NINT 14-28 NINT 7-28

**Supplementary Figure 1|Representative coronal slices and segmentation.** CT-derived coronal slices at end-expiration of representative animals (one per group) at each time point. The corresponding segmentation masks of the left (*green*) and right (*red*) lungs were over-imposed.


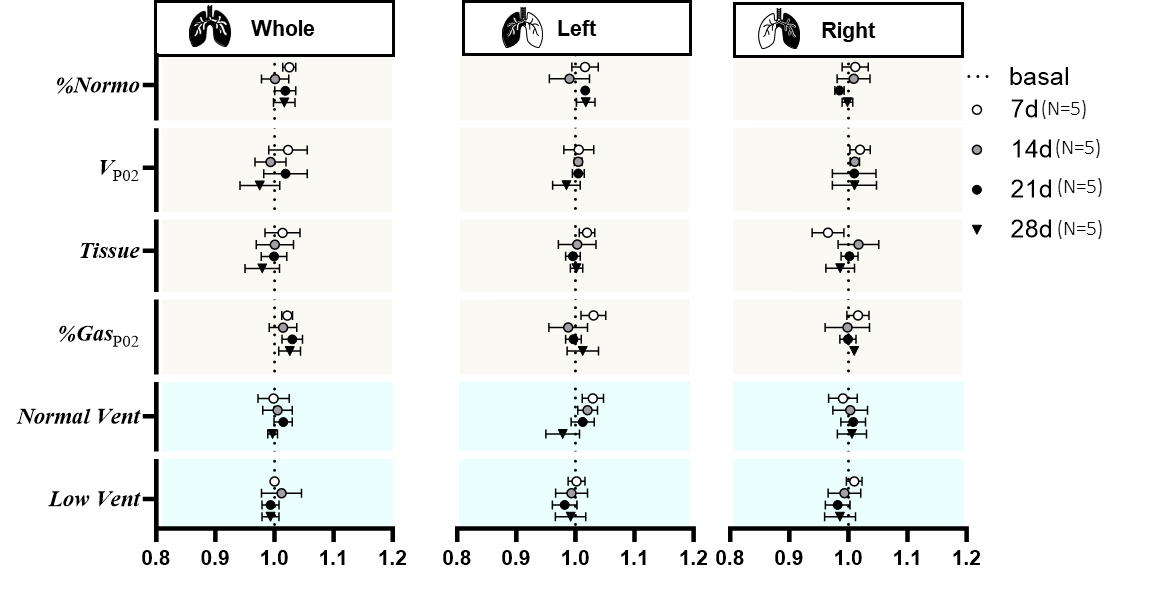


**Supplementary Figure 2| Micro-CT derived biomarkers in saline controls vs. basal condition.** All values are mean fold-changes with respect to the basal condition. 7d (*white dots*), 14d (*gray dots*), 21d (*black dots*), and 28d (*triangles*) data were expressed as mean ± SEM%. The dotted line at x=1 represents the basal condition (day 0). %Non and %Fibrosis are not shown, here, since they are equal to 0 in healthy animals. No statistically significant difference was revealed by one-way ANOVA followed by Dunnett’s t post-hoc test.


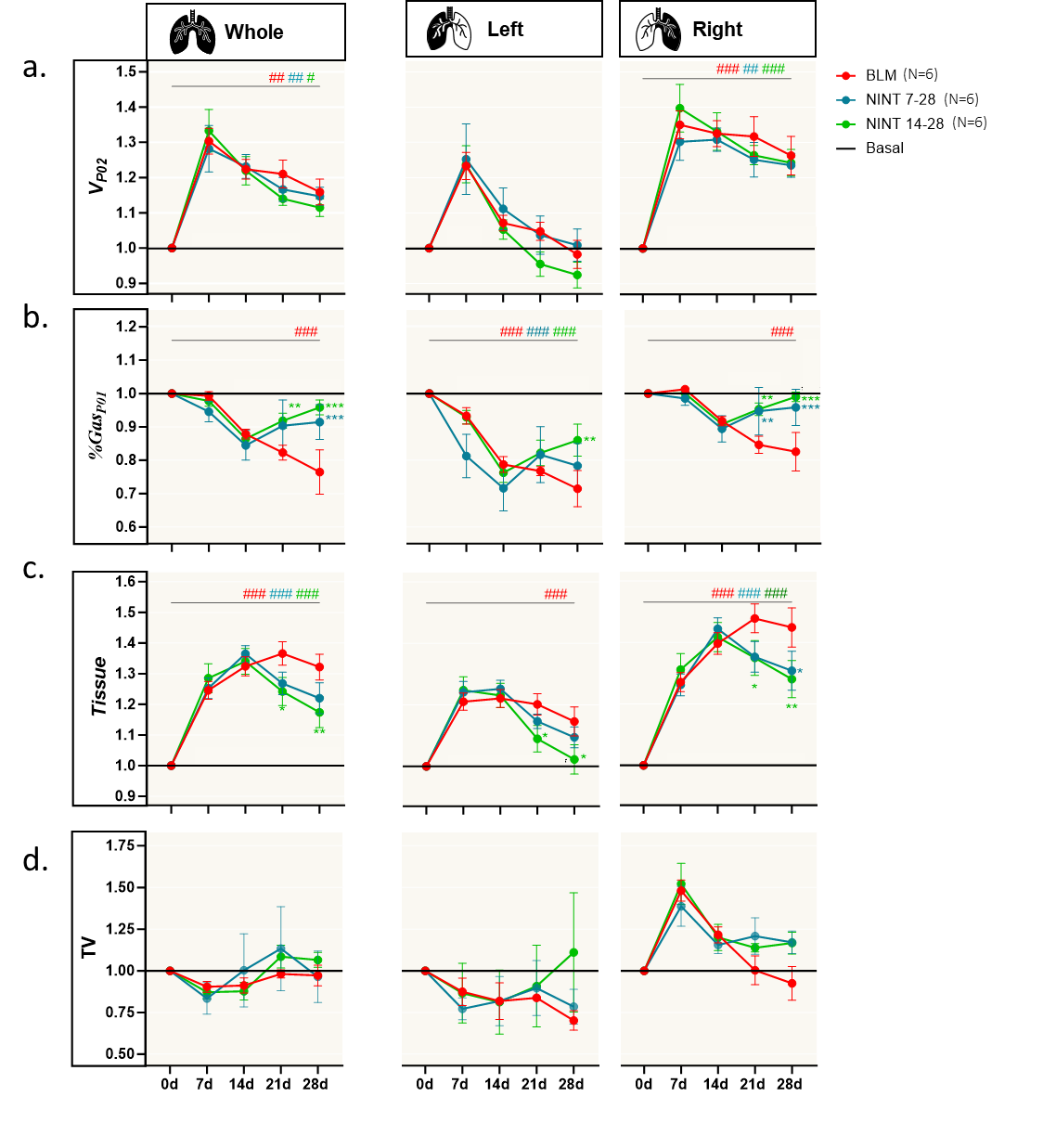


**Supplementary Figure 3| Automatically computed micro-CT-derived biomarkers.** The results of longitudinal quantifications of (a.) VP02, (b.) %GasP01, (c.) Tissue and (d.) TV are presented as fold-changes with respect to the basal condition, indicated by the horizontal *black line* at 1.0. BLM (*red*), NINT 7-28 (*blue*), and NINT 14-28 (*green*) raw data were expressed as mean ± SEM. Statistical significance of the longitudinal changes revealed by micro-CT-derived parameters in the different groups relative to the basal condition was assessed by two-way ANOVA followed by Šidák post-hoc test (#p < 0.05; ##p < 0.01; ### p < 0.001 0d vs. 28d). Statistical significance of the differences between the NINT and the BLM groups was calculated by two-way ANOVA followed by Dunnett’s t post-hoc test (*p < 0.05; **p < 0.01; ***p < 0.001 vs. BLM group).


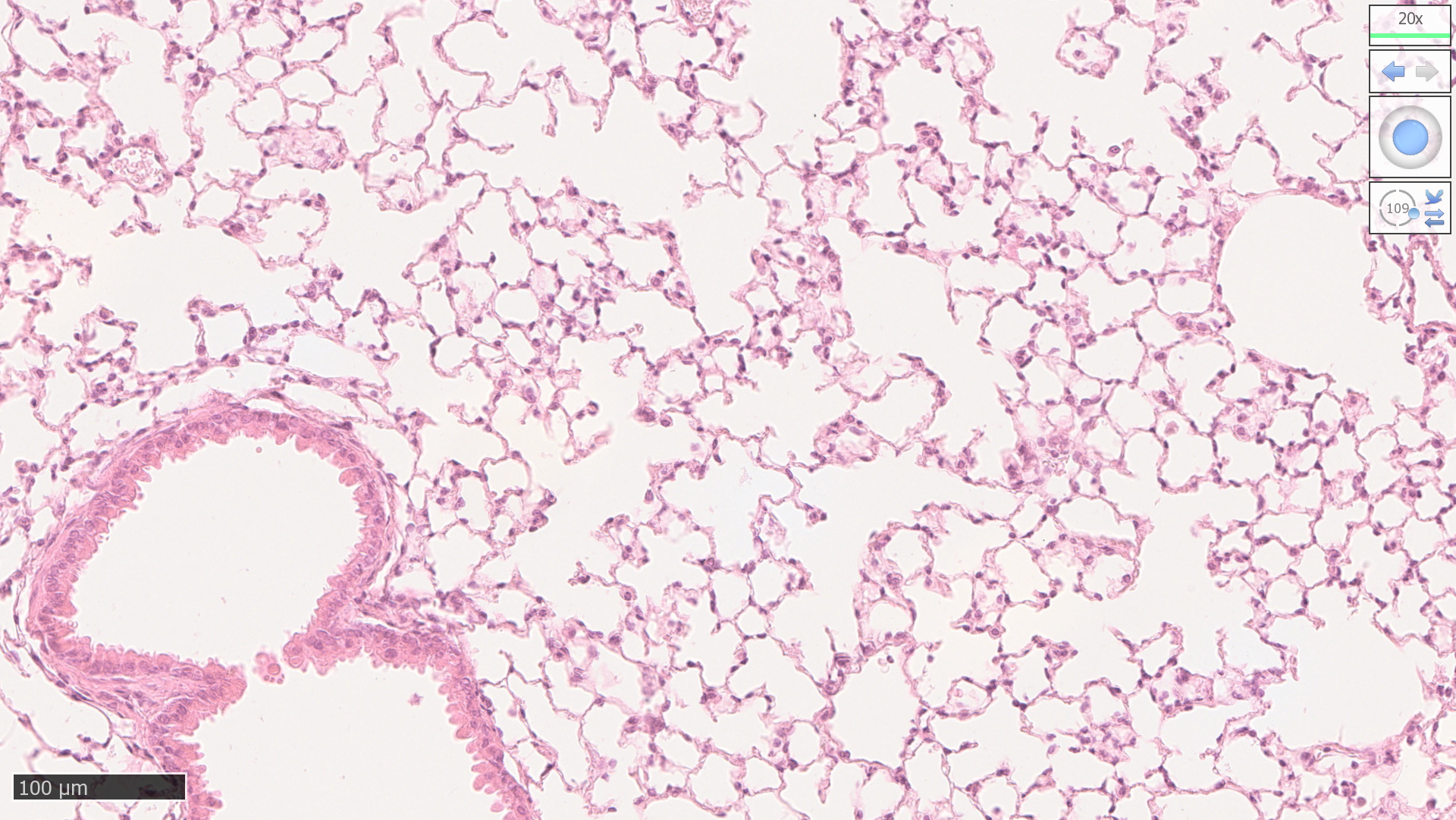

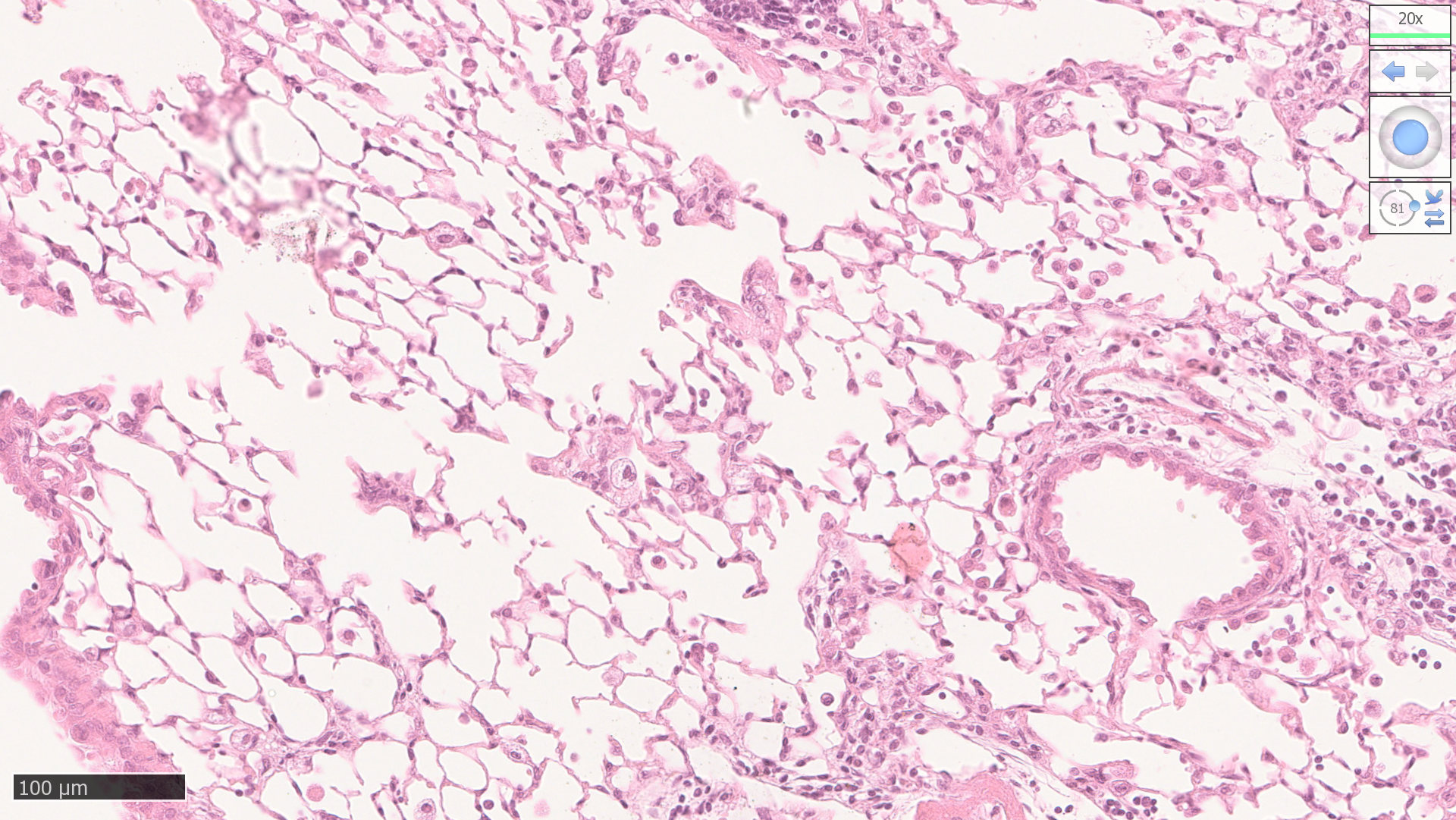

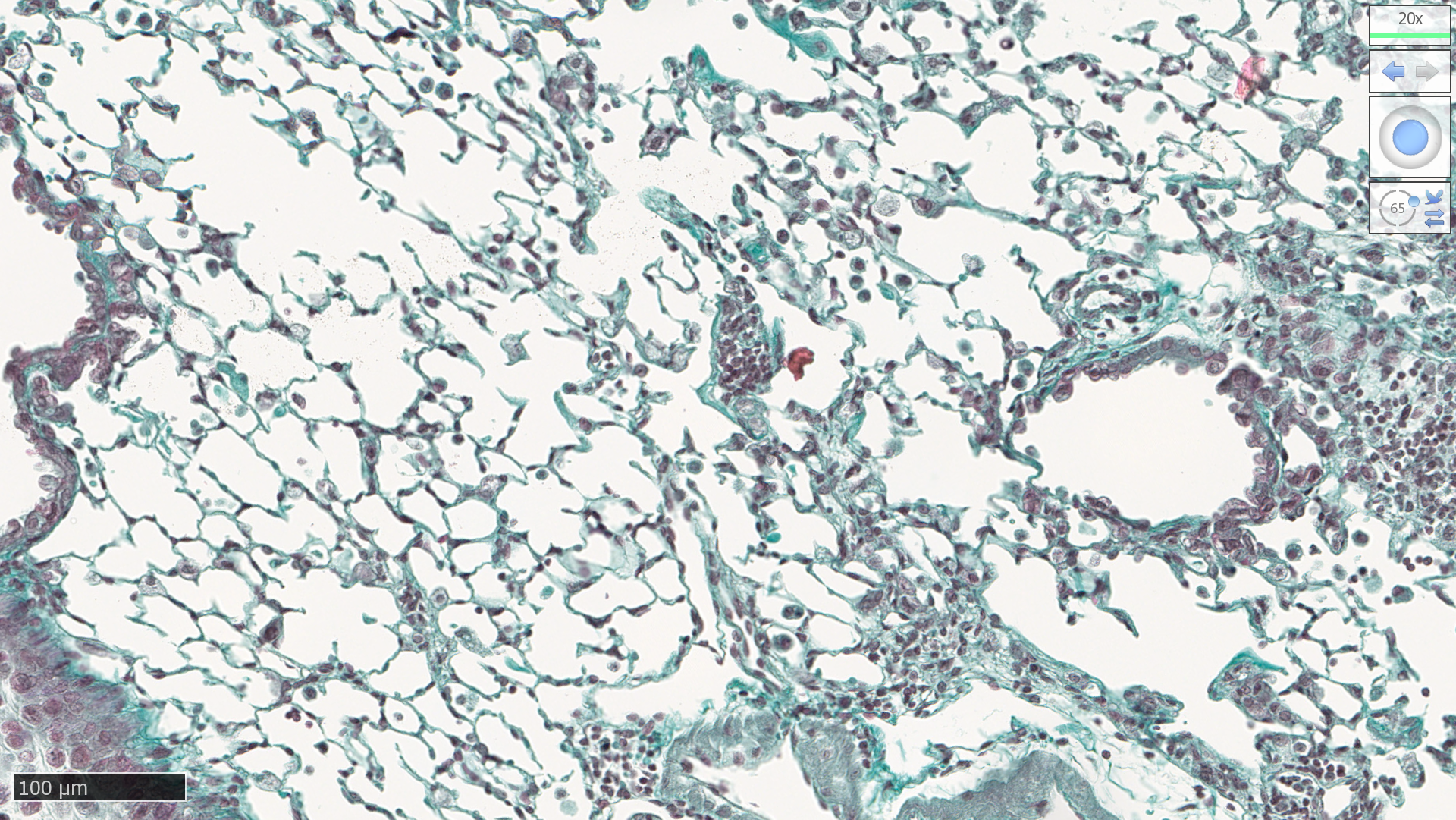

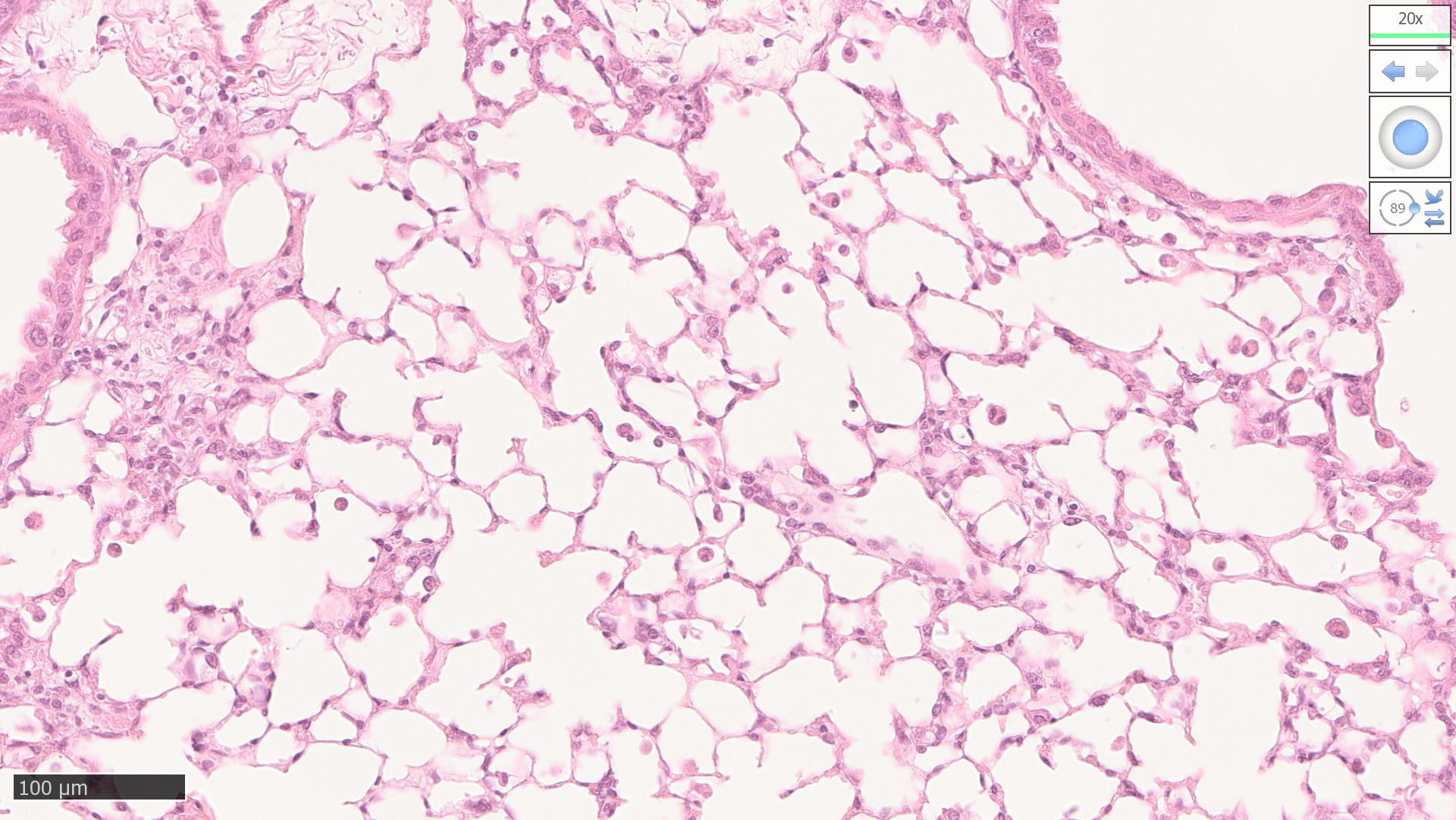

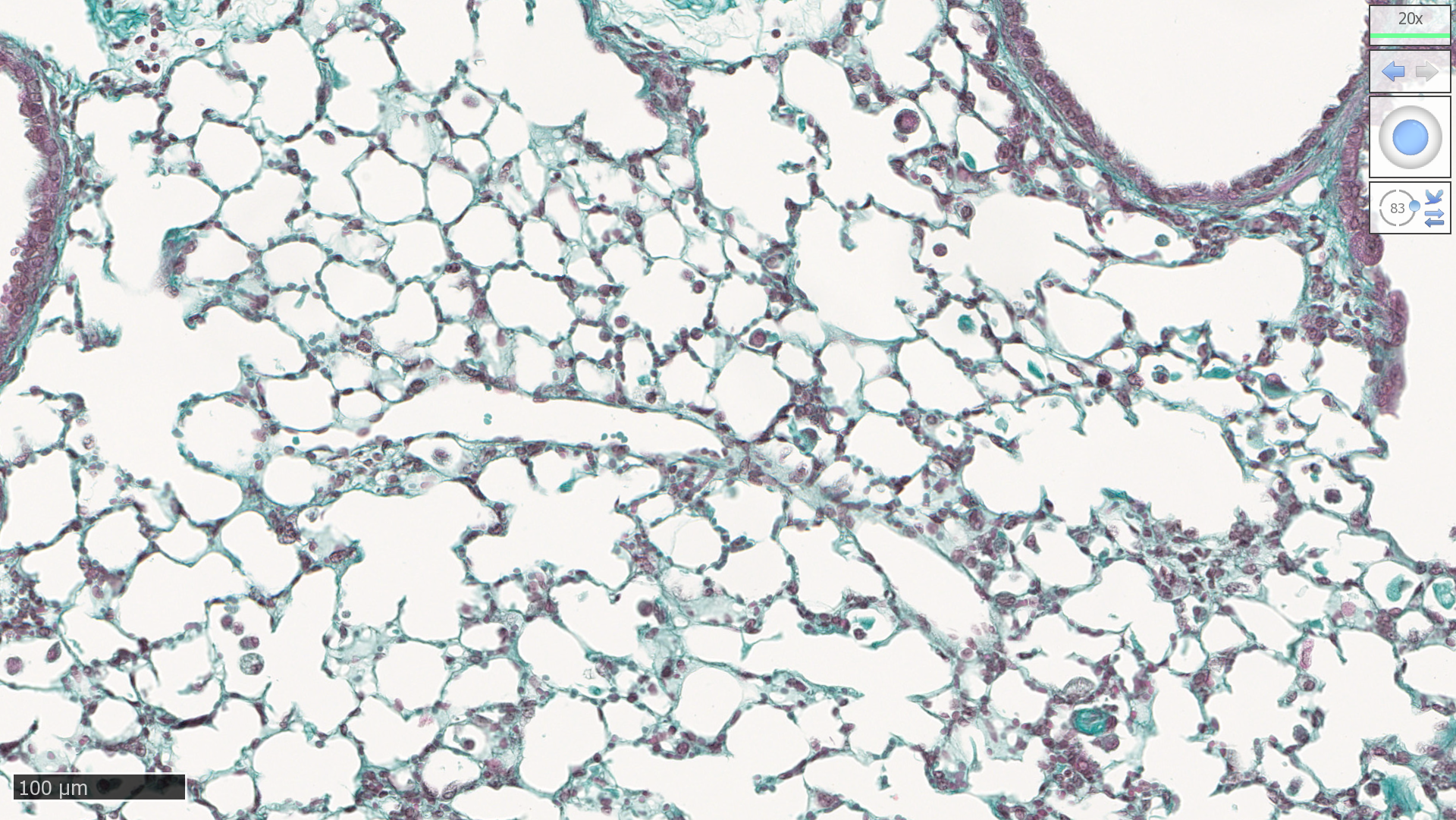

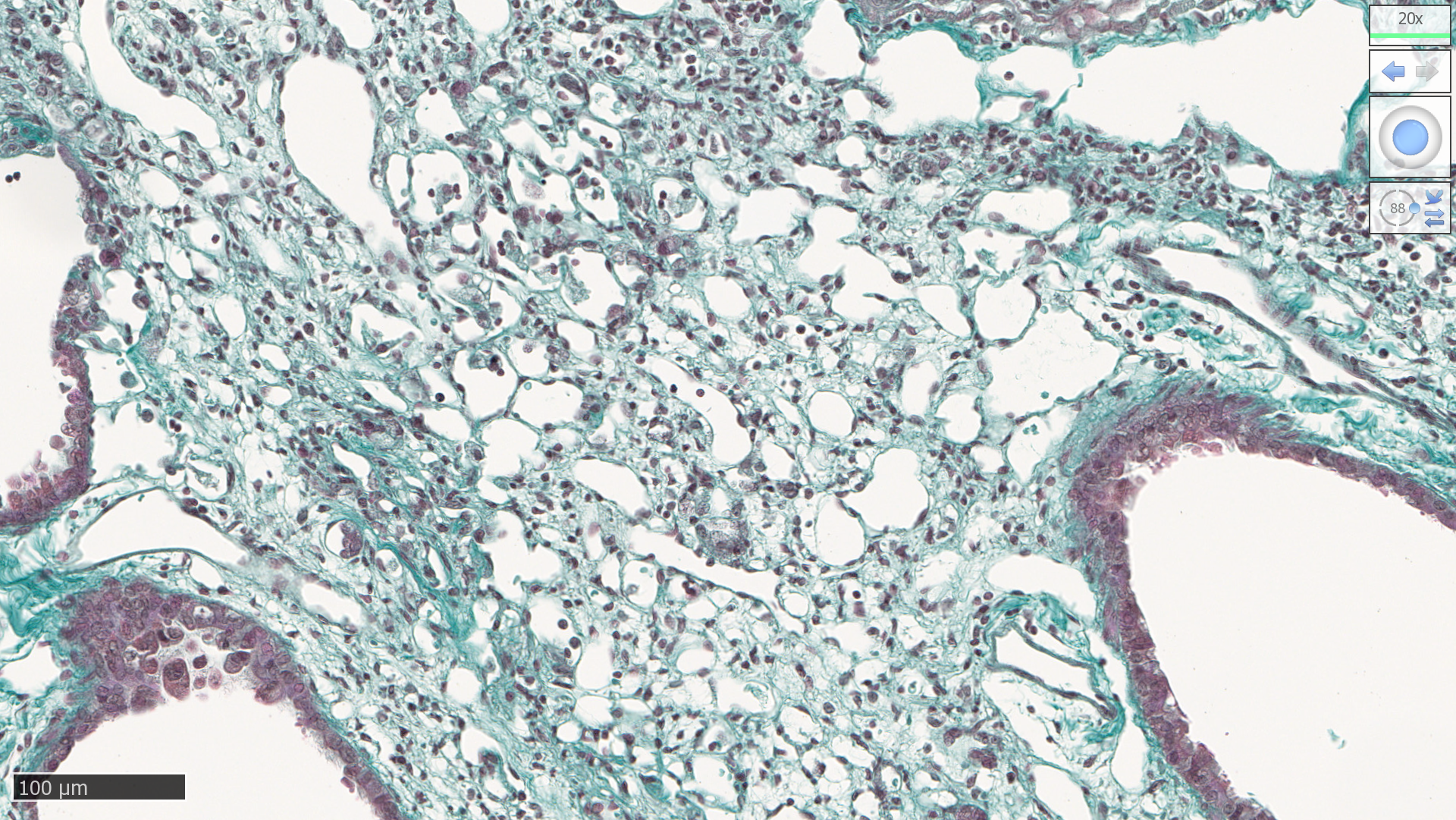

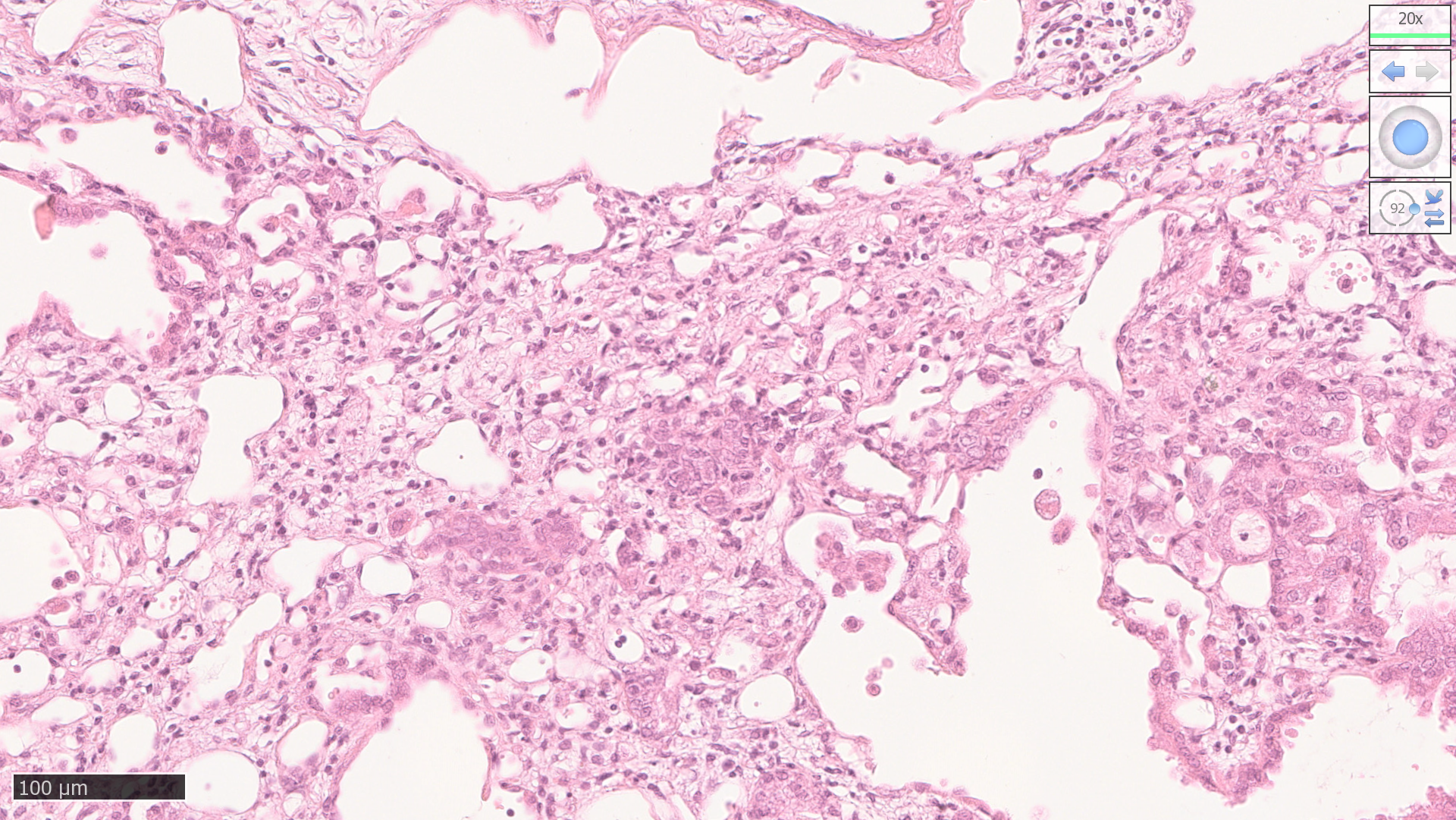

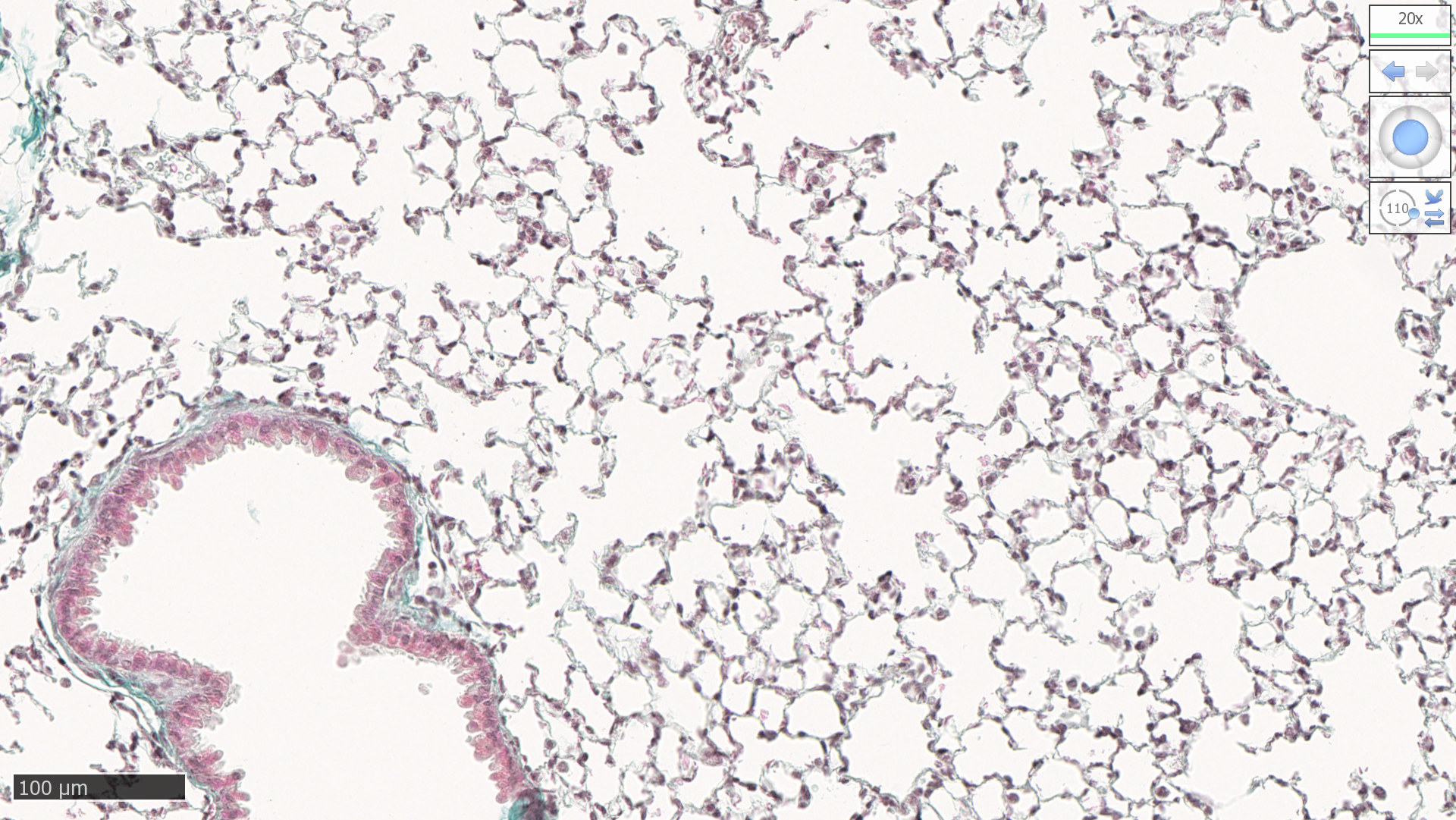

$$\boldsymbol{5 mm}$$

$$\boldsymbol{5}\boldsymbol{mm}$$

$$\boldsymbol{100}\boldsymbol{\mu m}$$

$$\boldsymbol{100}\boldsymbol{\mu m}$$

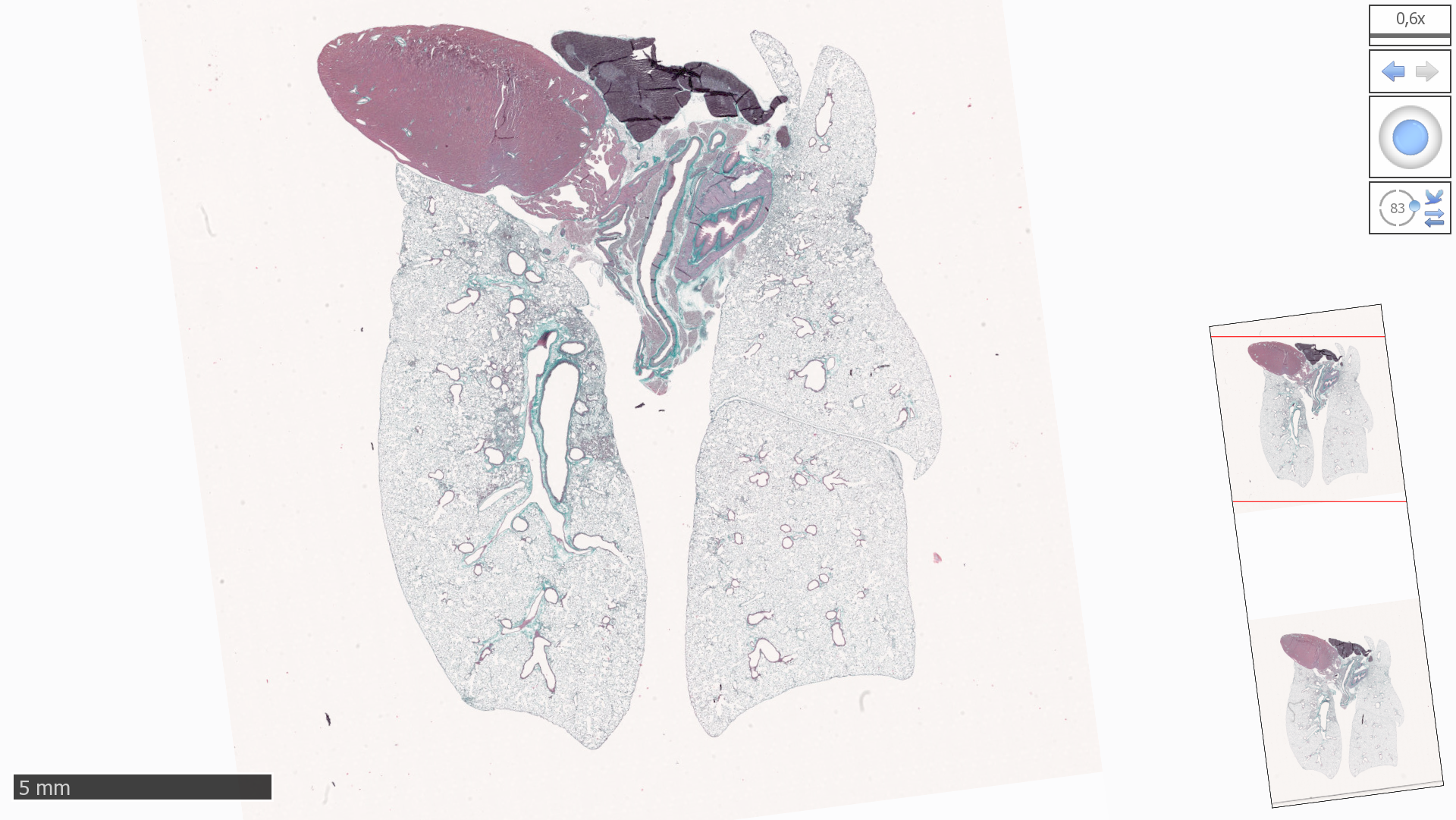

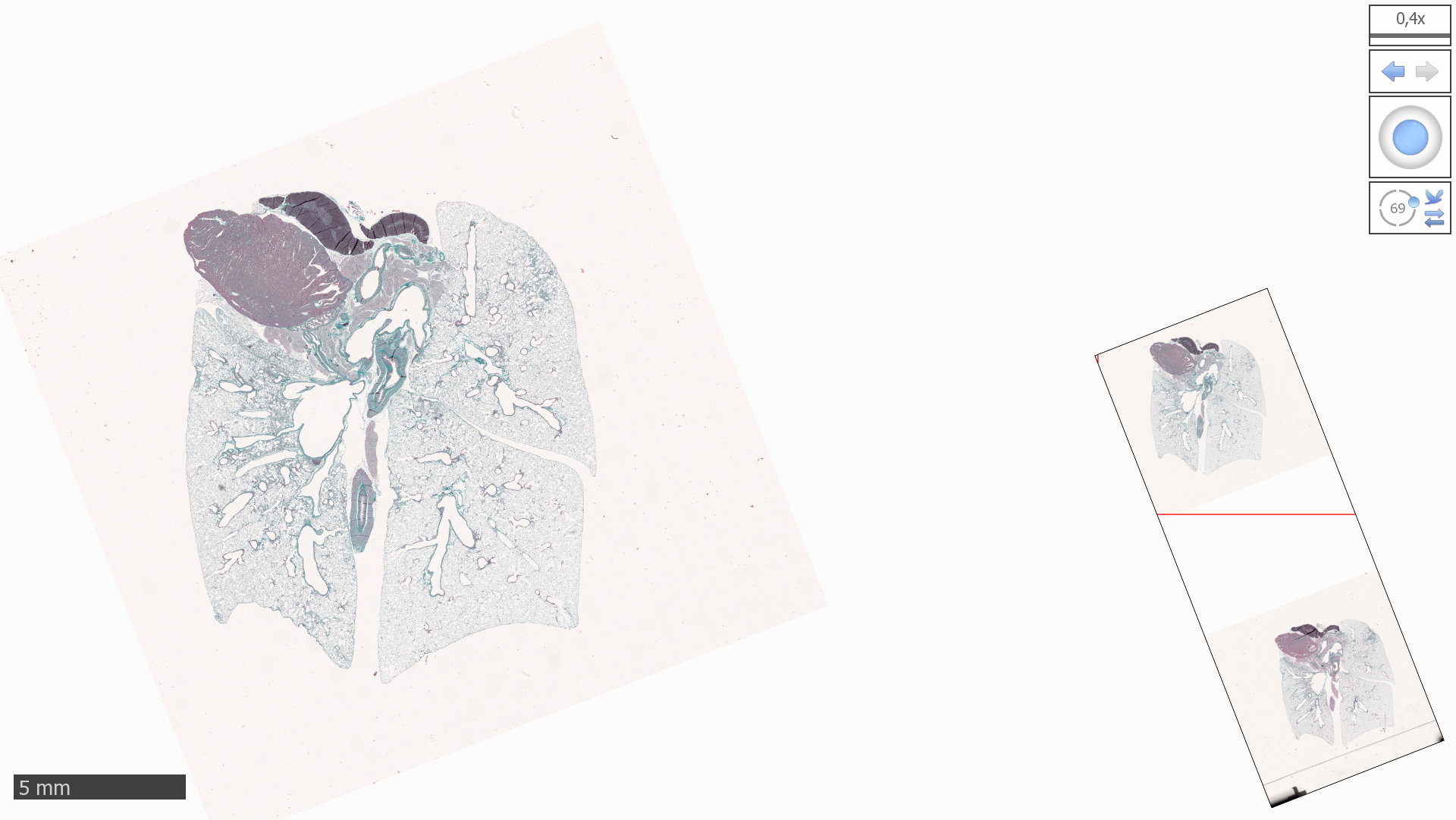

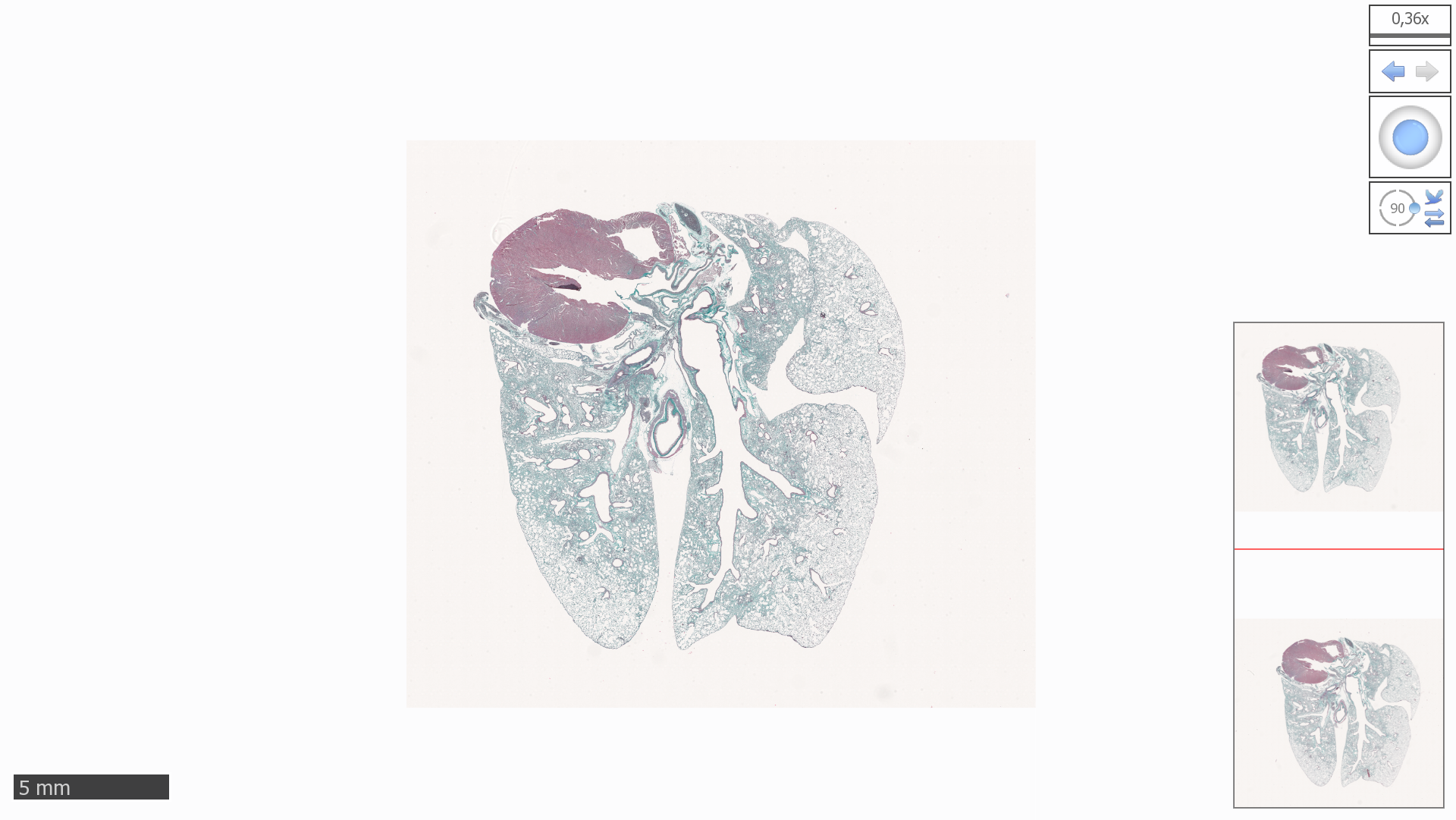

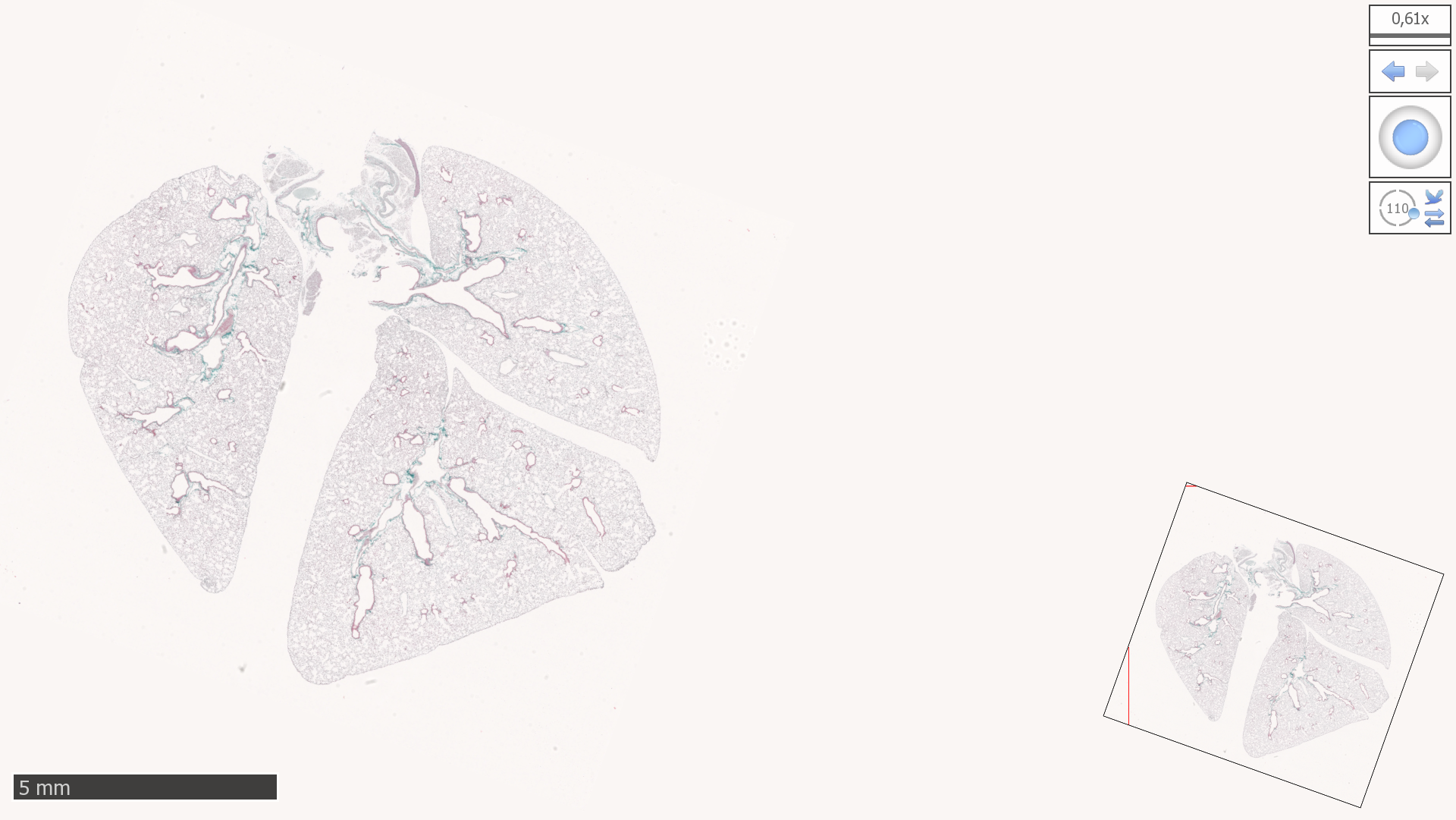


SAL

BLM

NINT 14-28

NINT 7-28

5 mm

5 mm


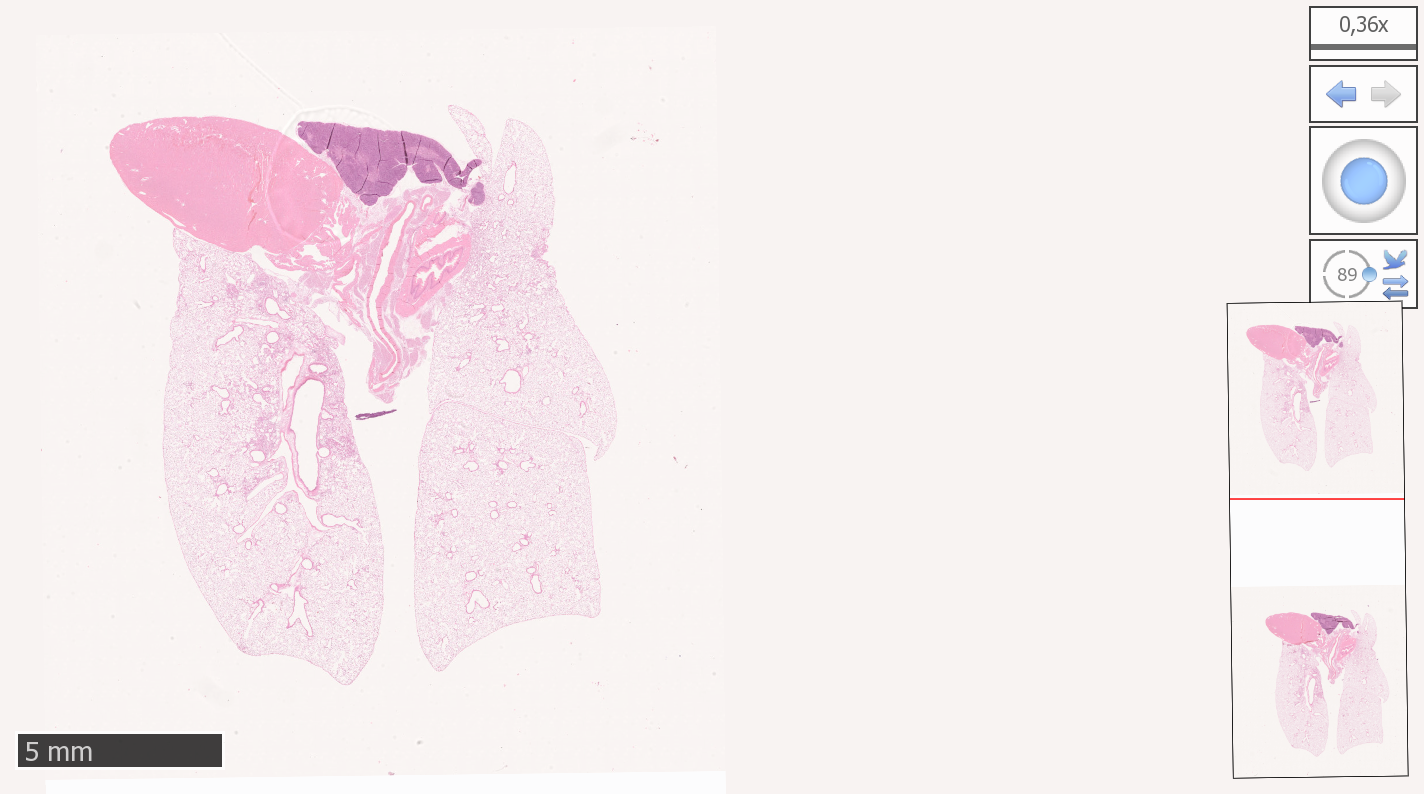

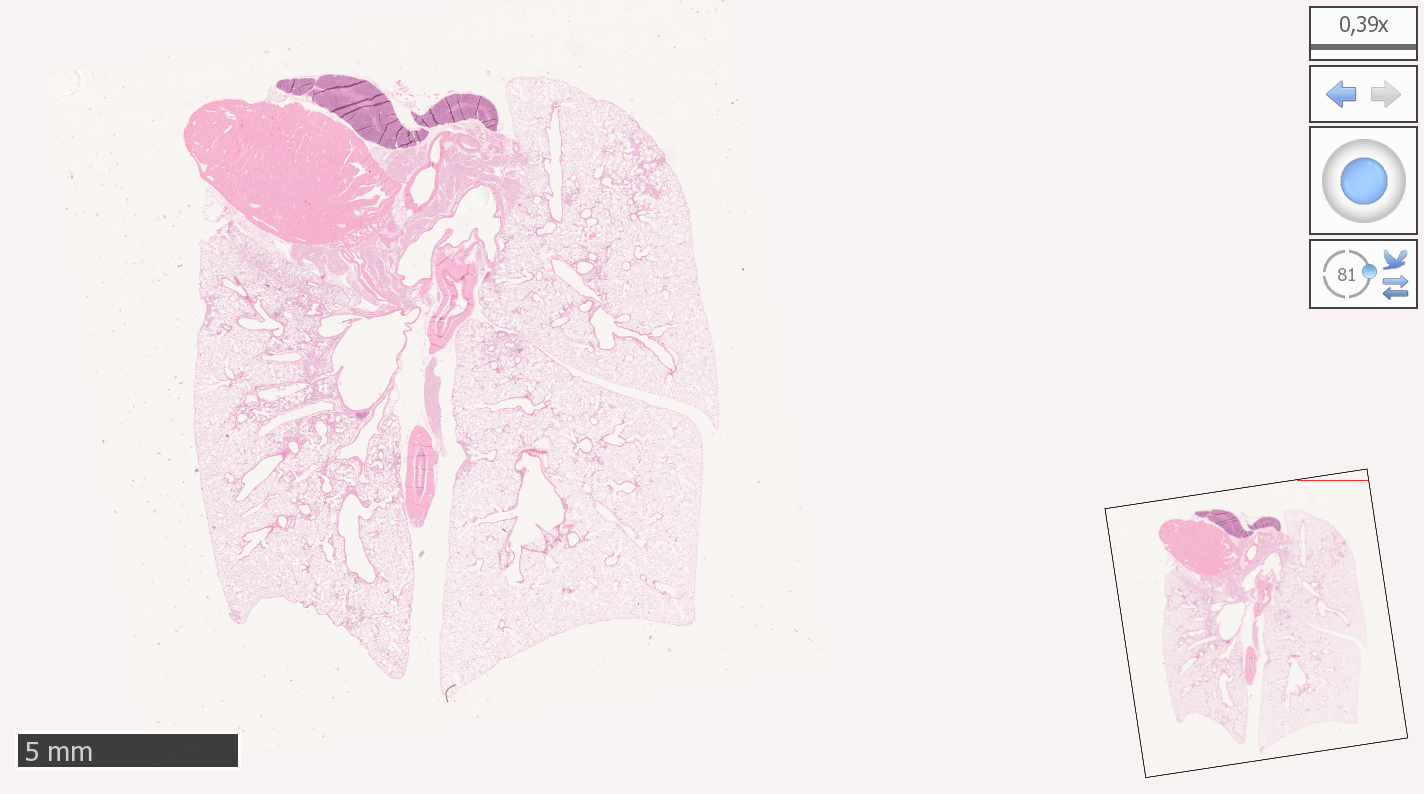

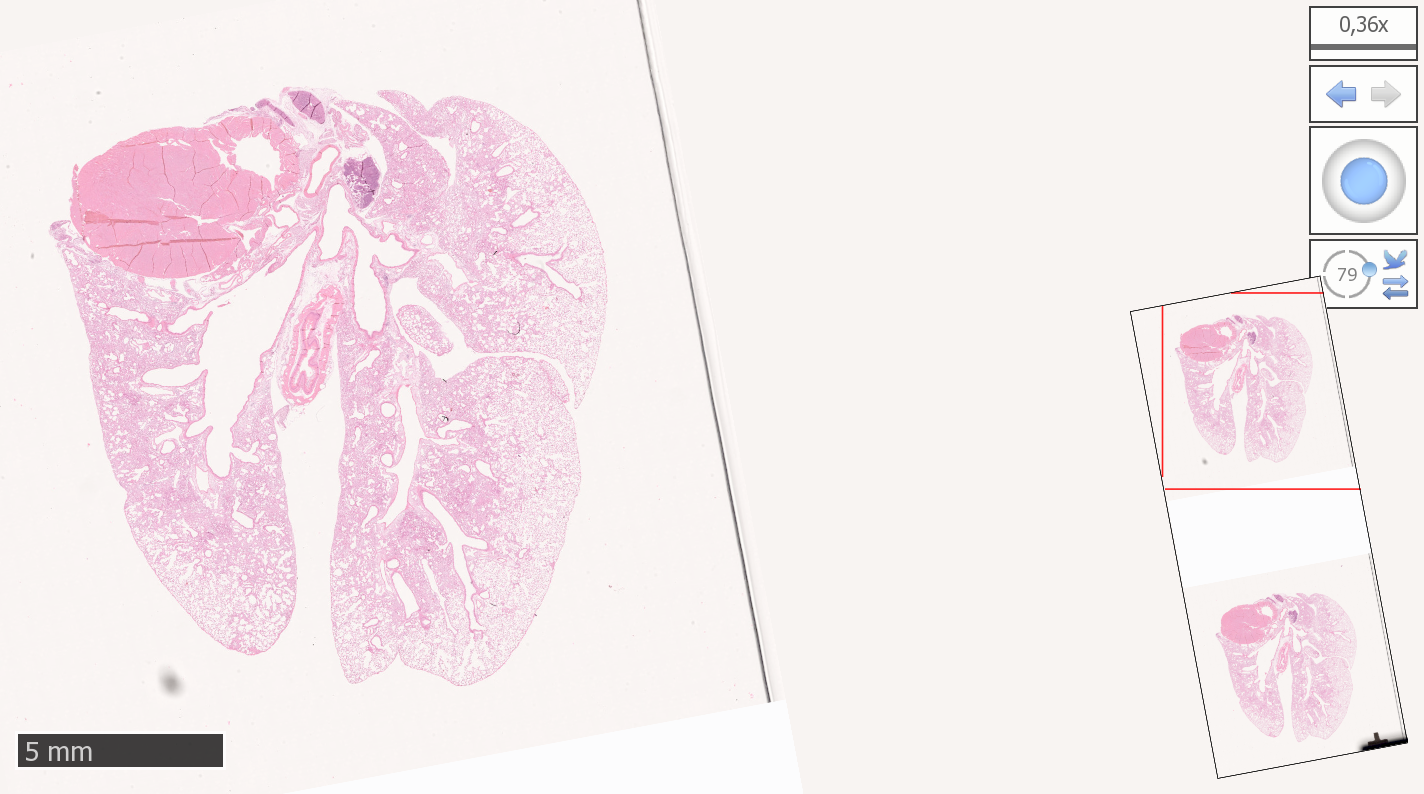

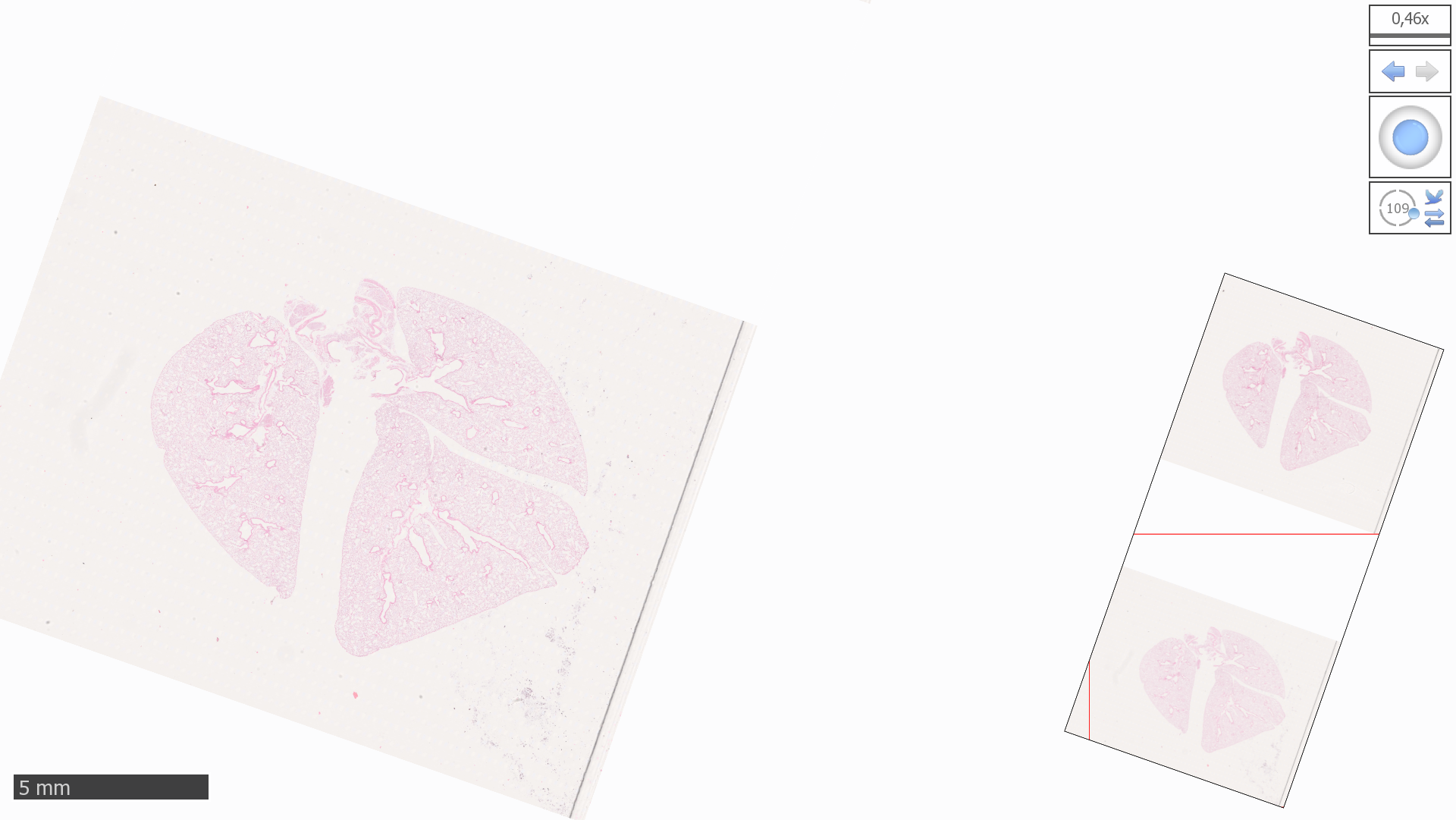


100 µm

100 µm

5 mm

a.

b.

**Supplementary Figure 4| Ex-vivo FFPE lung tissue.** a) Representative histological samples stained with hematoxylin and eosin (H&E): Above, whole-lung sections from all the indicated experimental groups at day 28, rectangles indicate fields reported below with higher magnification (20X); b) Representative histological samples stained with Masson's Trichrome (MT): Above, whole-lung sections from all the indicated experimental groups at day 28, rectangles indicate fields reported below with higher magnification (20X). Fibrotic loci are marked by *red* arrows.
