# Supplementary material for: Micro-CT-assisted identification of the optimal time-window for antifibrotic treatment in a bleomycin mouse model of long-lasting pulmonary fibrosis
Source: Sci Rep. 2024 Jun 26;14:14792. doi: 10.1038/s41598-024-65030-3 (PMC11208517; doi:10.1038/s41598-024-65030-3)
Supplement: Supplementary file 2 — Supplementary Tables. [file 41598_2024_65030_MOESM2_ESM.docx]

| micro CT readouts | | | | |
| --- | --- | --- | --- | --- |
|  | **Name** | **Description** | **Unit** | **Formula** |
| From P01 | $N_{P01}$ | Number of voxels at the end of inspiration phase (P01) | \ | Counting of lung voxels in P01 |
|  | $V_{P01}$ | Total lung volume at the end of inspiration phase (P01) | mm^3^ | $N_{P01}\cdot voxel size$ |
|  | ${MLA}_{P01}$ | Mean lung attenuation at the end of inspiration phase (P01) | HU | ${\sum_{i=1}^{N_{P01}} \left( HU \right)_{i}}/{N_{P01}}$ |
|  | Air | Volume of air at the end of inspiration phase (P01) | mm^3^ | $\frac{V_{P01}\cdot\mathrm{MLA}_{P01}}{-1000(HU)}$ |
| From P02 | $N_{P02}$ | Number of voxels at the end of expiration phase (P02) | \ | Counting of lung voxels in P02 |
|  | $V_{P02}$ | Total lung volume at the end of expiration phase (P02) | mm^3^ | $N_{P02}\cdot voxel size$ |
|  | ${MLA}_{P02}$ | Mean lung attenuation at the end of expiration phase (P02) | HU | ${\sum_{i=1}^{N_{P02}} \left( HU \right)_{i}}/{N_{P02}}$ |
|  | FRC | Functional residual capacity: Volume of air at the end of inspiration phase (P02) | mm^3^ | $\frac{V_{P02}\cdot\mathrm{MLA}_{P02}}{-1000(HU)}$ |

| micro CT biomarkers of interest | | | | |
| --- | --- | --- | --- | --- |
| Name | **Description** | **Unit** | **Formula** |  |
| $\boldsymbol{\%}\boldsymbol{Gas}_{\boldsymbol{P}\boldsymbol{02}}$ | Percentage of gas volume at the end of expiration phase (P02) | % | $FRC\cdot100/V_{P02}$ |  |
| Tissue | Lung volume without gas | mm^3^ | $V_{P02}-FRC$ |  |
| Tidal Volume (TV) | Volume of air exchanged between inspiration and expiration | mm^3^ | $Air-FRC$ |  |

**Supplementary Table 1**: micro CT automatically extracted parameters

**Supplementary Table 2**: micro CT automatically computed biomarkers of interest
